# Effect of tricyclic 1,2-thiazine derivatives in neuroinflammation induced by preincubation with lipopolysaccharide or coculturing with microglia-like cells

**DOI:** 10.1007/s43440-022-00414-8

**Published:** 2022-09-21

**Authors:** Benita Wiatrak, Edward Krzyżak, Berenika Szczęśniak-Sięga, Marta Szandruk-Bender, Adam Szeląg, Beata Nowak

**Affiliations:** 1 Department of Pharmacology, Faculty of Medicine, Wroclaw Medical University, ul. Mikulicza-Radeckiego 2, 50-345 Wroclaw, Poland; 2 Department of Inorganic Chemistry, Wroclaw Medical University, Wroclaw, Poland; 3 Department of Medicinal Chemistry, Wroclaw Medical University, Wrocław, Poland

**Keywords:** Alzheimer’s disease, Neurodegeneration, SH-SY5Y cells, In silico studies, Inflammation

## Abstract

**Background:**

Alzheimer’s disease (AD) is considered the most common cause of dementia among the elderly. One of the modifiable causes of AD is neuroinflammation. The current study aimed to investigate the influence of new tricyclic 1,2-thiazine derivatives on in vitro model of neuroinflammation and their ability to cross the blood–brain barrier (BBB).

**Methods:**

The potential anti-inflammatory effect of new tricyclic 1,2-thiazine derivatives (TP1, TP4, TP5, TP6, TP7, TP8, TP9, TP10) was assessed in SH-SY5Y cells differentiated to the neuron-like phenotype incubated with bacterial lipopolysaccharide (5 or 50 μg/ml) or THP-1 microglial cell culture supernatant using MTT, DCF-DA, Griess, and fast halo (FHA) assays. Additionally, for cultures preincubated with 50 µg/ml lipopolysaccharide (LPS), a cyclooxygenase (COX) activity assay was performed. Finally, the potential ability of tested compounds to cross the BBB was evaluated by computational studies. Molecular docking was performed with the TLR4/MD-2 complex to assess the possibility of binding the tested compounds in the LPS binding pocket. Prediction of ADMET parameters (absorption, distribution, metabolism, excretion and toxicity) was also conducted.

**Results:**

The unfavorable effect of LPS and co-culture with THP-1 cells on neuronal cell viability was counteracted with TP1 and TP4 in all tested concentrations. Tested compounds reduced the oxidative and nitrosative stress induced by both LPS and microglia activation and also reduced DNA damage. Furthermore, new derivatives inhibited total COX activity. Additionally, new compounds would cross the BBB with high probability and reach concentrations in the brain not lower than in the serum. The binding affinity at the TLR4/MD-2 complex binding site of TP4 and TP8 compounds is similar to that of the drug donepezil used in Alzheimer's disease. The ADMET analysis showed that the tested compounds should not be toxic and should show high intestinal absorption.

**Conclusions:**

New tricyclic 1,2-thiazine derivatives exert a neuroregenerative effect in the neuroinflammation model, presumably via their inhibitory influence on COX activity and reduction of oxidative and nitrosative stress.

**Supplementary Information:**

The online version contains supplementary material available at 10.1007/s43440-022-00414-8.

## Introduction

Neurodegenerative diseases have become a constantly growing socioeconomic burden and a medical problem in our aging population. The most common form of dementia in the elderly is Alzheimer’s disease (AD) [1]. Brains of patients affected with AD show intracellular amyloid-β deposits (senile plaques) with lowered Aβ_1-42_/Aβ_1-40_ ratio in cerebrospinal fluid and neurofibrillary tangles (NFTs) in extracellular space comprised of hyperphosphorylated *tau* proteins [2]. Chronic neuroinflammation with microglia and astrocyte activation accompanies those changes leading to neuronal death, synapse loss and progressive cognitive decline [3]. Risk factors for AD, besides age, include: carrying the ApoE ε4 allele, obesity, hypertension and type 2 diabetes (components of the metabolic syndrome) [4, 5].

For the last few decades, research on AD development and treatment was based mostly on the amyloid cascade hypothesis [6]. All of the observable changes are secondary to the Aβ burden caused by impaired enzymatic cleavage of the amyloid protein precursor (APP) and ineffective clearance of its deposits. Overproduction of aggregation-prone Aβ_1-42_ leads to the formation of toxic oligomers and later fibrils in the form of characteristic senile plaques. Later, microglia and astrocytes are activated, and tauopathy follows [7]. As a consequence of this approach, most developed and clinically tested therapies focused on the depletion of amyloid deposits, oligomers and monomers [8]. Unfortunately, the results of the trials show that even the complete elimination of Aβ did not improve cognitive functions in patients undergoing this treatment. Moreover, some serious side effects were linked to these therapies, such as micro hemorrhage and increased risk of infections in the central nervous system (CNS) [9, 10]. To further denote the amyloid as the main AD triggering agent, no correlation has been found between Aβ burden and clinical manifestation of the disease [1].

The inefficacy of the pursued model of treatment led to the surprising conclusion that amyloid-β, considered as a purely pathological hallmark of the disease, might play a role in maintaining the homeostasis of the brain. Since then, Aβ has been found to possess antibacterial and antifungal properties both in vitro and in vivo [11, 12]. The similarity of molecular structure and ability to self-aggregate, bind to microbial cell walls and immobilize the pathogenes support the inclusion of amyloid-β in the family of antimicrobial peptides (AMPs) [4, 9, 13]. In line with this assumption, Aβ production and aggregation is not a cause of AD but merely an exacerbated response reaction to the presence of external, dangerous agents. Research shows that the blood–brain barrier (BBB) permeability increases with age, allowing various potentially harmful elements to enter the brain [1]. Senile plaques have been found to include bacterial particles like lipopolysaccharide (LPS) entrapped in their structure [14]. Moreover, the brains of patients with AD have higher concentrations of LPS than the healthy age-matched control group [15]. Chronic and repeating *Porphyromonas gingivalis* infections or changes in gut microbiota have also been linked to an increased risk of Alzheimer’s disease [1, 16].

Recently, more and more attention has been brought to understanding the inflammatory aspect of neurodegeneration, which usually precedes the onset of AD and may play a central role in its progression [3, 17]. Neuroinflammation is a complex phenomenon affecting multiple pathways and employing various types of cells inhabiting the CNS. However, the main representatives of the immune system in the immunopriviliged brain are microglia and astrocytes. Unlike the beneficial short-term reaction that neutralizes dangerous agents, the chronic activation of microglia cells in AD causes the overproduction of pro-inflammatory cytokines (IL-1β, IL-6, TNF-α), oxidative stress, impairment phagocytosis, neuronal damage and synapse loss [18–20]. Microglia acquire its active state upon stimulation of toll-like receptors (TLRs) 2 or 4, and both amyloid-β and LPS can interact with those surface proteins, perpetuating and exacerbating the inflammation of the brain [21, 22]. Furthermore, microglia mediate the astrocyte neurotoxic activation leading to neuronal death [20]. Cellular debris, reactive oxygen or nitrogen species (ROS, RNS) and pro-inflammatory cytokines stimulate the innate immunological reaction in CNS even after eliminating the primary signal [23].

Mitigating the harmful and damaging neuroinflammation could potentially slow the progression of the disease, alleviate some of the symptoms or even prevent the onset of AD in patients at risk [23]. This study aimed to investigate the anti-inflammatory properties of new tricyclic 1,2-thiazine derivatives in SH-SY5Y cells differentiated to the neuronal phenotype incubated with either a bacterial lipopolysaccharide or supernatant from microglial THP-1 cell culture. One compound with a bicyclic structure was also included in the study to investigate the effect of the presence of the third ring on cellular activity. The studied 1,2-benzothiazine tricyclic derivatives differ among themselves in the size of the third ring, which is six, seven or eight-membered. Moreover, electron-donating (CH_3_ or OCH_3_) or electron-withdrawing (Br or Cl) groups were introduced into their structure to determine their influence on the properties of the compound. Thiazine compounds exhibit a wide range of pharmacological activities, including antibacterial, antifungal, antimalarial, antineoplastic, antiviral, anti-inflammatory, and analgesic properties. Moreover, the anti-inflammatory effect of thiazine alkaloids is mediated by the inhibition of superoxides. Therefore, they may be an alternative to NSAIDs in the future, as it has been shown that they do not irritate the gastrointestinal tract at doses showing anti-inflammatory and analgesic effects [24].

MTT assay was used to assess cell viability by evaluating mitochondrial metabolic activity. DCF-DA assay measured the oxidative response, and the Griess assay tested the production of nitric oxide. In turn, fast halo assay (FHA) determined the level of DNA damage.

## Materials and methods

### Cell lines

Human neuroblastoma SH-SY5Y (CRL-2266) and monocytic leukemia THP-1 (TIB-202) cell lines were both obtained from American Type Culture Collection (ATCC; Manassas, VA, US). Incubation with retinoic acid (RA; Sigma-Aldrich, Saint Louis, MO, US; cat. no. R2625) induces differentiation of SH-SY5Y cells to a suitable neuronal model. Furthermore, THP-1 cultures stimulated with phorbol-12-myristate-13-acetate (PMA; Sigma-Aldrich; cat. no. P8139) acquire morphological and functional similarity to macrophages and therefore can serve as a model of microglia—the so-called macrophages of the brain.

Culture conditions were identical for both cell lines. The incubator maintained the 37 °C and 5% CO_2_ concentration in humidified air. Cells were passaged twice a week. The medium was removed from SH-SY5Y cultures, and they were incubated with TrypLE Express solution (Gibco, Thermo Fisher Scientific, Waltham, MA, US; cat. no. 12604-021) for 5 min at 37 °C. The obtained cell suspension was transferred to a centrifugal tube and complemented with the complete medium to a 1:1 volume ratio. Cells were centrifuged (1000×*g*, 5 min), the supernatant was removed, and fresh medium was added. THP-1 as cells growing in suspension were not incubated with trypsin during passaging but only transferred to a centrifugal tube and treated like SH-SY5Y cell line from this moment.

### Culture media

Growth medium for the SH-SY5Y cell line contained MEM (Modified Eagle Medium) supplemented with 10% fetal bovine serum (FBS; Biological Industries, Nahariya, Israel; cat. no. 04-001-1A), 2 mM l-glutamine (Lonza, Basel, Switzerland, cat. no. 17-605E), 25 μg/ml gentamicin (Lonza; cat. no. 17-518L) and 2.5 μg/ml amphotericin B (Gibco; cat. no. 15290026). For differentiation, the content of FBS was reduced to 2.5%, and retinoic acid in a concentration of 10 μM was added. The medium was changed every other day for 5 days.

THP-1 cells were grown in a medium comprised of RPMI-1640 (Lonza; cat. no. BE12-702F) supplemented with 10% fetal bovine serum (FBS; Biological Industries; cat. no. 04-001-1A), 2 mM l-glutamine (Lonza, cat. no. 17-605E), 25 μg/ml gentamicin (Lonza; cat. no. 17-518L) and 2.5 μg/ml amphotericin B (Gibco; cat. no. 15290026). The differentiation medium was prepared by adding 5 ng/mL PMA (phorbol 12-myristate 13-acetate) to the growth medium.

### Tested compounds

New tricyclic 1,2-thiazine derivatives (TP1, TP4, TP5, TP6, TP7, TP8, TP9, TP10) were obtained from the Department of Medicinal Chemistry, Wroclaw Medical University. The method of synthesis and studies confirming the structures of these compounds are described in the article of Maniewska et al. [25]. Complete structures of all eight compounds tested are presented in Table 1. The compounds were dissolved in DMSO to a stock concentration of 10 mM and stored at − 20 °C. Immediately before applying the compounds, the tested concentrations in the range of 10–100 µM were prepared by dissolving in the growth medium.

**Table 1:**
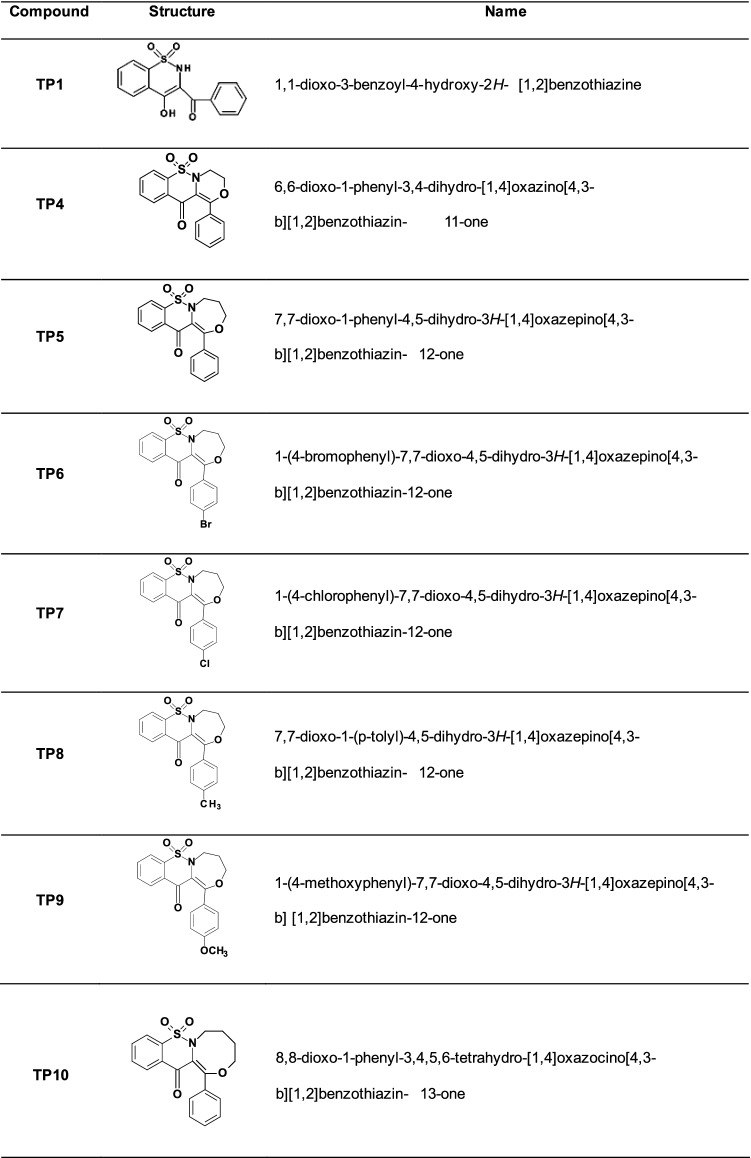
Structures and names of tested compounds

LPS was purchased from Sigma-Aldrich, and concentrations of 5 µg/ml and 50 µg/ml were used in the study.

### Experimental design

Bioassays were performed in 96-well (MTT, DCF-DA, Griess) or 24-well (FHA) culture plates with cells seeded at a density of 10,000 or 50,000 cells per well, respectively. After 24 h of incubation in the growth medium, SH-SY5Y and THP-1 cells were induced to differentiate with RA or PMA, respectively, for 5 days. Subsequently, 100 µl of LPS or 30 µl of microglia culture supernatant mixed with 70 µl of growth medium was added for another 24 h. The medium was then removed, and the wells were washed with PBS. Finally, tested tricyclic 1,2-thiazine derivatives were added for a 24-h incubation. Then biological assays were performed—measurements of cell viability (evaluation of the mitochondrial metabolic activity in MTT assay) and the levels of oxidative stress (DCF-DA), nitrite ions production (Griess), and double-stranded DNA damages (FHA).

In the preliminary study of the effects of tested compounds on SH-SY5Y cells without the use of harmful agents, one control was used—culture without tested derivatives. In the second phase of the study, where preincubation with LPS or microglia supernatant was used, two controls without tested compounds were applied—negative control preincubated with a harmful factor, and positive control to which no toxic agent (LPS or microglia supernatant) was added.

### MTT assay

The MTT assay was used to assess the cells’ viability by spectrophotometric measurement of the concentration of purple formazan—a product of active mitochondrial metabolism. First, 1 mg/mL solution was prepared by dissolving MTT in MEM. Then, the supernatant was removed from cultures, and wells were filled with MTT solution and plates were incubated for 2 h at 37 °C. After that, the supernatant was removed and replaced with 100 μL of isopropanol for 30 min to dissolve the formed formazan crystals. Finally, the absorbance at 570 nm was measured with a Varioskan LUX microplate reader (Thermo Fisher Scientific, Waltham, MA, US).

### DCF-DA assay

The reactive oxygen species (ROS) level was determined in the DCF-DA assay. First, the reagent for testing was obtained by diluting the ethanol DCF-DA solution in PBS at a ratio of 1:1000. Then, the supernatant was removed from the tested cultures, and 100 μL of DCF-DA solution was added. After 1 h of incubation at 37 °C, ROS level was measured using a Varioskan LUX microplate reader with excitation at 485 nm and emission at 535 nm.

### Griess assay

Griess assay was used to measure the production of nitric oxide (NO) in cells. The Griess solution is a mixture of 1% sulfanilamide in 5% phosphoric acid and 0.1% *N*-(1-Naphtyl)ethylenediamine dihydrochloride in a 1:1 volume ratio. Supernatant from cultures (50 μL) was transferred to a clean plate and incubated with 50 μL of Griess reagent for 20 min at RT protected from light. Absorbance was measured at 548 nm with a Varioskan LUX microplate reader.

### Fast halo assay (FHA)

The fast halo assay served as a tool to assess the damage of the nuclear DNA in the form of double-strand breaks (DSBs). Supernatant from cell cultures was collected to separate centrifugal tubes. Cells were detached from wells using TrypLE solution (5 min incubation at 37 °C) and transferred to corresponding tubes. Next, plates were washed with Hanks’ Balanced Salt Solution (HBSS) to collect the remaining cells. Tubes were centrifuged (1000×*g*, 5 min), and the supernatant was replaced with fresh HBSS twice. Collected cell pellets were placed in a warm (37 °C) water bath and mixed with 130 μL of 1.25% low melting point agarose. Glass slides pre-coated with a high melting point agarose were then covered with the created cell suspensions, closed with coverslips and put on a cooling block for about 10 min. Later, the coverslips were removed, and the slides stayed overnight in a lysis buffer at 4 °C. Then, they were transferred to alkaline buffer (pH = 13) for 30 min and washed with neutralizing buffer twice for 5 min. Finally, slides were stained with 5 μL 4′,6-diamidino-2-phenylindole (DAPI) dye for 20 min in the dark and photographed using a fluorescence microscope (Leica Microsystems, Wetzlar, Germany) with a 40 × objective lens.

The analysis was performed for 5 independent replicates. Cells were harvested from the wells, and one slide was prepared for each concentration of each tested compound. The entire slide was analyzed by capturing images of cell nuclei. For each replicate, 10 randomly selected nuclei were evaluated.

Micrographs of cell nuclei were analyzed using proprietary software specially prepared for this purpose. The application is based on the OpenCV library and its task is to facilitate and speed up the analysis of large amounts of HALO images. Using the application, the operator measures the diameter of the cell nucleus and the diameter of the nuclear halo (chromatin dispersion). The ratio of the nucleus diameter to the halo, which corresponds to the degree of DNA damage, is then automatically calculated.

### Total cyclooxygenase (COX) activity

To measure cyclooxygenase (COX) activity, COX Activity Assay Kit (Cayman Chemical, Ann Arbor, MI, US; cat. no. 760151) was used. Cyclooxygenase peroxidase activity was measured by a colorimetric method for determining oxidized *N*,*N*,*N*′,*N*′-tetramethyl-p-phenylenediamine (TMPPD). After treatment with the test compounds, the cell cultures were mechanically separated from the surface of the multiwell plates. Next, cells were centrifuged and resuspended in cold buffer (0.1 M Tris–HCl, pH = 7.8 containing 1 mM EDTA) for homogenization and centrifuged again at 10,000×*g* for 15 min at 4 °C. Then, the collected supernatant was used for testing. All samples were evaluated in triplicate to assess COX-1, COX-2, and total COX activity.

### In silico calculations

Molecular descriptors: hydrogen-bond acidity (A) and basicity (B), polarizability (S), molar refraction (E), McGowan volume (V) of a solute, polar surface area (PSA) were evaluated by PaDEL-Descriptor open-source software [26]. KOWWIN v1.68 (EPI Suite; U.S. Environmental Protection Agency, Washington, DC, US) calculated logP. The free energy of solvation (Gsolv) after geometry optimization (using density functional theory (DFT) with Becke’s three-parameter hybrid exchange function with the Lee–Yang–Parr gradient corrected correlation (B3LYP) functional in combination with 6-311+G (d,p) basis set) was carried out using the Gaussian 2016 A.03 software package. The probability of crossing the blood–brain barrier was calculated using the AdmetSAR web tool [27].

The crystal structure of TLR4/MD-2 complex (3VQ2) was obtained from Protein Data Bank (http://www.rcsb.org). The structure of the studied compounds was optimized using DFT functional with B3LYP/6-311+G (d,p) basic set. All the ligands and water molecules were removed. and then polar hydrogen atoms and Kollman charges were added to the protein structure using AutoDock Tools 1.5.6 [28]. To prepare the ligand molecules partial charges were calculated, and nonpolar hydrogens were merged. and rotatable bonds were assigned. The centre of the grid box was set according to the binding pocket site in the crystal structure. The size of the search space was selected to be 30 × 30 × 30 Å. The molecular docking study was conducted using AutoDockVina 1.1.2 [29]. Exhaustiveness values were set as 8, 16, 24, and 60. After the molecular docking. the ligand-receptor complexes were further analyzed using Discovery Studio Visualizer v.20 (https://www.3ds.com/).

### Statistical analysis

For MTT, DCF-DA and Griess assays, 5 independent experiments were performed involving a 5-well study at each concentration. Only the COX activity was evaluated in triplicate. The results are presented as the *E*/*E*_0_ ratios, where *E* is the mean value of the parameter tested, and *E*_0_ is the mean of the control (negative if two controls were used in a given case). The scatter of the results is marked in the form of standard deviation. The normality of data distribution and the equality of variance was checked for all the obtained results. The Shapiro–Wilk test confirmed the normal distribution of the data, and Levene’s test the equality of the variance. Hence, all analyzes were carried out using parametric tests. The one-way analysis of variance (one-way ANOVA) and Tukey’s post-hoc method was used, when appropriate, in the statistical analyzes performed using Statistica 13 software (Dell Software Inc., Round Rock, TX, USA). The results of the ANOVA are given as the F-statistic and the degrees of freedom associated with it. The significance point was set at *p* = 0.05. The graphs indicate the significance against the negative and positive control. All figures were prepared using an Excel application (Microsoft Inc., Albuquerque, NM, US).

MCDA analysis was performed for tested derivatives at the lowest concentration of 10 µM using the weighted sum model in the Excel application. However, the COX activity test performed only for 100 µM concentration was an exception. Hence, the results for this concentration were included in the MCDA. The analysis took covered the differences in the results obtained for the tested compound compared to the negative control with 50 µg/ml LPS. The weights used in the analysis were selected to standardize the significance of individual tests (based on the maximum values of differences), except the COX-1 level, for which the weight was 2 times lower than for the other tests.

## Results

### Effect of tested compounds on SH-SY5Y cell line

**Fig. 1:**
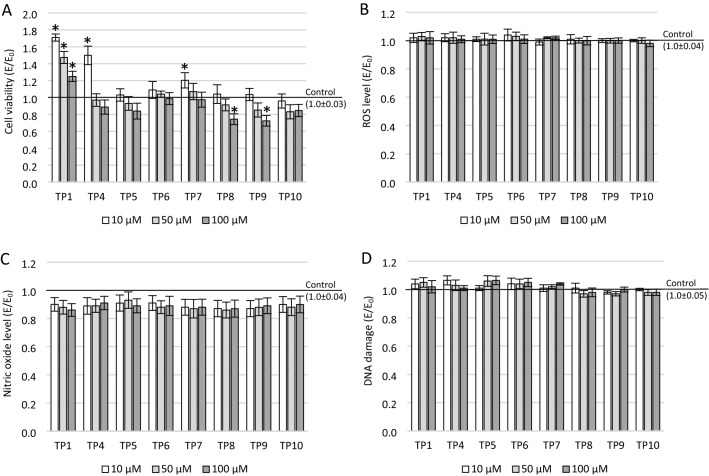
The effect of tested compounds on SH-SY5Y cells: **A** cell viability measured in MTT assay, **B** reactive oxygen species (ROS) level measured in DCF-DA assay, **C** nitric oxide (NO) level measured in Griess assay, **D** DNA damage measured in fast halo assay (FHA); *p ≤ 0.05—significant difference compared to the control without tested compounds. Presented data are mean ± SD of *n* = 5 independent experiments and were analyzed by one-way ANOVA with Tukey’s post hoc test

**Fig. 2:**
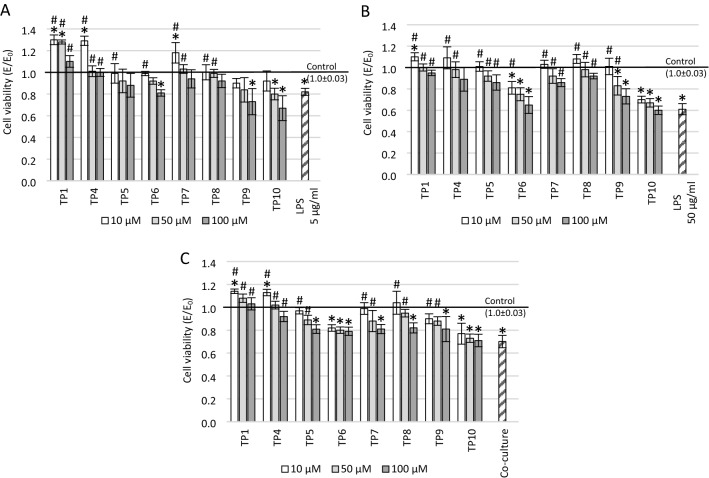
The effect of tested compounds on cell viability measured in MTT assay. The SH-SY5Y neuronal cells were preincubated with: **A** 5 μg/ml lipopolysaccharide (LPS), **B** 50 μg/ml LPS, **C** supernatant from THP-1 cell culture; *p ≤ 0.05—significant difference compared to positive control without tested compounds, LPS or supernatant; ^#^*p* ≤ 0.05—significant difference compared to negative control with a harmful agent and without tested compounds. Presented data are mean ± SD of *n* = 5 independent experiments and were analyzed by one-way ANOVA with Tukey’s post hoc test

The effect on cell viability in the MTT assay of nearly all compounds tested (except TP10) was dose-dependent. The mean viability of cell cultures differed between the studied groups: *F*_24_ = 45.02, *p* < 0.001 (Fig. 2A). It is worth emphasizing that all tested compounds at concentrations of 10 and 50 μM did not significantly reduce the metabolic activity of mitochondria. Most of the compounds at the highest concentration (100 µM) also did not cause a significant decrease in cell culture viability (except for TP8 and TP9—*p* < 0.001). On the other hand, TP1 in the entire studied concentration range (*p* < 0.001), 10 μM TP4 (*p* < 0.001), and 10 μM TP7 (*p* = 0.02) significantly induced the proliferation of neuronal cells (Fig. 1A). All compounds in the tested concentration range did not affect the level of free oxygen radicals (*F*_24_ = 1.54, *p* = 0.12; Fig. 1B). In the Griess test, the compounds (TP1, TP4-TP10) did not significantly reduce the production of nitric oxide in neuronal cells (*F*_24_ = 1.31; *p* = 0.18; Fig. 1C).The one-way ANOVA showed statistical significands in DNA strand breaks (DBSs) between groups—*F*_24_ = 7.369, *p* < 0.001. The level of damage for any of the tested compounds was not statistically significantly different from the control (Fig. 1D).

### Effect of tested compounds on cell viability in tested conditions

The one-way ANOVA showed a significant main effect of treatment on the viability of cells preincubated in 5 μg/ml LPS (*F*_25_ = 60.10517, *p* < 0.001), 50 μg/ml LPS (*F*_25_ = 40.21536, *p* < 0.001), and also cells preincubated with supernatant from THP-1 cells (*F*_25_ = 48.70848, *p* < 0.001). In all investigated cases, the influence of tested compounds on cell viability evaluated in the MTT assay was dose-dependent. Higher viability was noted at low concentrations, while a concentration of 100 µM turned out to be even harmful in the case of some tested derivatives.

In cultures pre-treated with 5 μg/ml LPS, 10 μM and 50 μM concentrations of TP1 (*p* < 0.001), as well as 10 μM of TP4 (*p* < 0.001) and TP7 (*p* = 0.02), significantly increased cell viability inducing neuronal proliferation (Fig. 2A)—in these cases, the metabolic activity was significantly higher even compared to the positive control without LPS. A statistically significant reduction in cell culture viability compared to the positive control was observed at a concentration of 100 µM of TP6 (*p* = 0.009), TP9 (*p* < 0.001), and at a concentration of 50 µM (*p* = 0.004) and 100 µM (*p* < 0.001) of TP10. Cell viability was higher at all tested concentrations for TP1 (*p* < 0.001) and TP4 (*p* < 0.001 for 10 µM, *p* = 0.01 for 50 µM and *p* = 0.02 for 100 µM) compared to the negative control with LPS. The compounds TP7 and TP8 showed a positive effect at concentrations of 10 µM (*p* < 0.001 for TP7; *p* = 0.02 for TP8) and 50 µM (*p* = 0.002 for TP7; *p* = 0.04 for TP8). In turn, compounds TP5 and TP6 positively impact cell viability only at 10 µM concentration (*p* = 0.043 for TP5; *p* = 0.047 for TP6). In contrast, TP9 and TP10 compounds at concentrations of 100 µM decreased cell viability below the negative control level (although this difference did not reach statistical significance).

In cultures preincubated with 50 μg/ml LPS, the lowest tested concentration of TP1, TP4, TP5, TP7, TP8, and TP9 increased cell viability to levels close to the control without LPS (Fig. 2B). In turn, at all concentrations of TP6 (*p* = 0.01 for 10 µM, *p* < 0.001 for 50 µM and 100 µM) and TP10 (*p* < 0.001), and at a concentration of 50 µM (*p* = 0.005) and 100 µM (*p* < 0.001) of TP9, cell viability was statistically significantly lower compared to the control without LPS. Compared to the negative control incubated with 50 µg/ml LPS, a significant increase in metabolic activity was observed for all concentrations of TP1, TP4, TP5, TP7 and TP8 derivatives (*p* < 0.001), 10 µM TP6 (*p* < 0.001), and 10 μM and 50 µM TP9 (*p* < 0.001).

In the co-culture model, compounds TP1 and TP4 at each tested concentration increased the viability compared to the control culture incubated with the microglia supernatant (*p* < 0.001; Fig. 2C). A regenerative effect on the viability of the culture was also observed at the concentrations of 10 µM and 50 µM of the compounds TP5 (*p* < 0.001), TP7 (*p* < 0.001), TP8 (*p* < 0.001) and TP9 (*p* < 0.001). In addition, 10 µM concentrations of compounds TP1 and TP4 significantly increased mitochondrial metabolic activity even compared to positive control without LPS (*p* = 0.01 for TP1; *p* = 0.04 for TP4). In the whole range of tested concentrations, statistically significantly lower cell viability was observed compared to the positive control for TP6 (*p* < 0.001) and TP10 (*p* < 0.001). In addition, lower viability of the culture compared to the positive control was noted at a concentration of 100 µM for the compounds: TP5 (*p* < 0.001), TP7 (*p* < 0.001), and TP8 (*p* < 0.001).

### Effect of tested compounds on ROS or NO in tested conditions

**Fig. 3:**
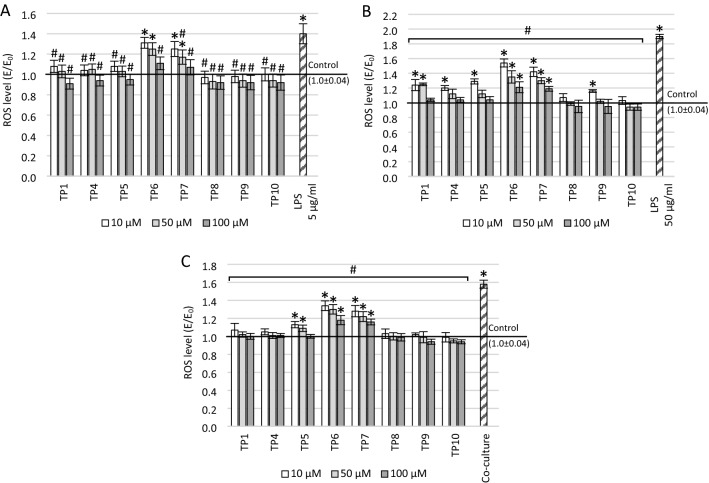
The effect of tested compounds on reactive oxygen species (ROS) level measured in DCF-DA assay. The SH-SY5Y neuronal cells were preincubated with: **A** 5 μg/ml lipopolysaccharide (LPS), **B** 50 μg/ml LPS, **c** supernatant from THP-1 cell culture; *p ≤ 0.05—significant difference compared to positive control without tested compounds, LPS or supernatant; ^#^*p* ≤ 0.05—significant difference compared to negative control with a harmful agent and without tested compounds (in cells preincubated with 50 μg/ml LPS or supernatant, statistical significance was obtained for each concentration of each tested compound). Presented data are mean ± SD of *n* = 5 independent experiments and were analyzed by one-way ANOVA with Tukey’s post hoc test

**Fig. 4:**
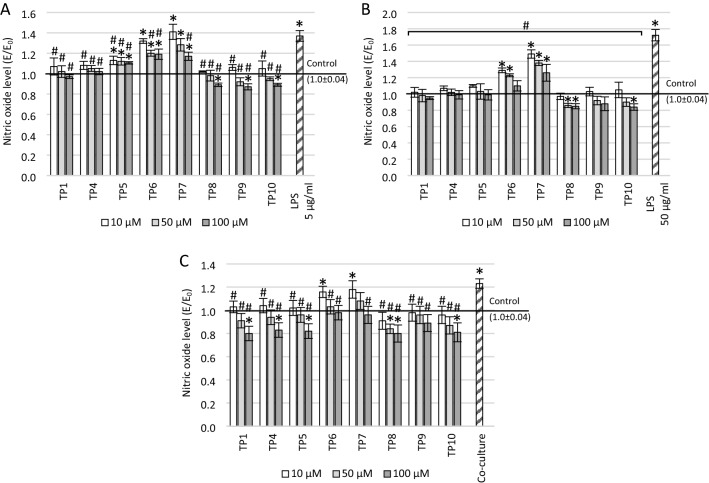
The effect of tested compounds on nitric oxide (NO) level measured in Griess assay. The SH-SY5Y neuronal cells were preincubated with: **A** 5 μg/ml lipopolysaccharide (LPS), **B** 50 μg/ml LPS, **c** supernatant from THP-1 cell culture; *p ≤ 0.05—significant difference compared to positive control without tested compounds, LPS or supernatant; #*p* ≤ 0.05—significant difference compared to negative control with a harmful agent and without tested compounds (in cells preincubated with 50 μg/ml LPS, statistical significance was obtained for each concentration of each tested compound). Presented data are mean ± SD of n = 5 independent experiments and were analyzed by one-way ANOVA with Tukey’s post hoc test

The levels of free oxygen radicals (ROS) and nitric oxide (NO) were assessed by the DCF-DA and Griess assays, respectively (Figs. 3, 4). Both LPS and co-culturing with THP-1 supernatant increased the levels of ROS and NO. Moreover, this increase for LPS was concentration-dependent—the higher the concentration, the higher the level of free radicals. Treatment of the cell cultures with each compound resulted in a concentration-dependent reduction in both ROS and NO levels (the higher the concentration, the stronger the free radical scavenging).

One-way ANOVA showed a significant main effect on ROS level in the cultures preincubated with 5 µg/ml LPS.

(*F*_25_ = 21.62; *p* < 0.001). Most of the compounds (except for TP6 and TP7) significantly reduced the ROS level compared to control with LPS (*p* < 0.001) to levels close to the positive control, while TP6 significantly reduced free radicals only at 100 µM (*p* < 0.001) and TP7 at 50 µM and 100 µM (*p* < 0.001; Fig. 3A). At 10 µM and 50 µM concentrations of TP6 (*p* < 0.001) and TP7 (*p* < 0.001 for 10 µM; *p* = 0.01 for 50 µM), ROS level after treatment with compounds remained significantly higher than in the positive control without LPS.

In cell cultures preincubated with a higher concentration of LPS (50 µg/ml; Fig. 3B), the significance of differences between groups in ANOVA was also noted (*F*_25_ = 87.80; *p* < 0.001). There has been a reduction in the ROS level for all compounds over the entire concentration range tested (*p* < 0.001). The level of free oxygen radicals close to the positive control was observed after incubation with TP1 at 100 µM and TP4, TP5, and TP9 at 50 µM and 100 µM. The TP8 and TP10 compounds reduced the ROS to the level of positive control at each tested concentration. Statistically significant differences in ROS levels compared to the positive control were observed for 10 µM and 50 µM of TP1 (*p* < 0.001), 10 µM TP4 (*p* < 0.001), 10 µM TP5 (*p* < 0.001), 10 µM TP9 (*p* < 0.001), and the entire concentration range of TP6 and TP7 (*p* < 0.001).

In co-culture, ANOVA also showed a significant main effect of treatment on the ROS level (*F*_25_ = 80.78; *p* < 0.001). The ROS level was significantly reduced for all compounds at each tested concentration compared to the control preincubated with the THP-1 supernatant (*p* < 0.001; Fig. 3C). The compounds TP1, TP4, TP8, TP9, and TP10 in the entire concentration range and TP5 at the concentration of 100 µM caused ROS reduction to the level of positive control. Compounds TP6 and TP7 (in the whole concentration range) and TP5 at a concentration of 10 and 50 µM reduced ROS, but their level was still higher than the positive control (*p* < 0.001 for TP6 and TP7; *p* < 0.001 for 10 µM TP5 and *p* = 0.048 for 50 µM TP5).

ANOVA indicated also a significant main effect on the level of nitric oxide in cultures pre-incubated with 5 µg/ml LPS (*F*_25_ = 81.66; *p* < 0.001). Most of the tested compounds (except TP6 and TP7) caused a statistically significant reduction in the NO level compared to the negative control incubated only with 5 µg/ml LPS in the entire concentration range tested (*p* < 0.001; Fig. 4A). TP6 induced a statistically significant NO reduction only at 50 µM and 100 µM (*p* < 0.001) and TP7 only at 100 µM (*p* < 0.001). In addition, the NO level at a concentration of 100 µM of TP8 (*p* = 0.03), TP9 (*p* = 0.002), and TP10 (*p* = 0.03) was significantly lower than the positive control. For all concentrations of TP1 and TP4 the NO level was close to the positive control. In the case of TP5, TP6, and TP7 compounds, the NO level was significantly higher than the positive control over the entire concentration range (*p* < 0.001).

In cell cultures preincubated with 50 µg/ml LPS (Fig. 4B), the significance of differences between groups in ANOVA was also observed (*F*_25_ = 87.26; *p* < 0.001). All compounds tested over the entire concentration range resulted in a reduction of the NO level. In most cases (except for 10 µM and 50 µM of TP6 and all TP7 concentrations; *p* < 0.001 in all cases), the NO level was close or lower than the positive control. Moreover, for 100 µM TP10 (*p* = 0.005) and 50 µM (*p* = 0.03) and 100 µM (*p* = 0.01) TP8, the NO level was statistically lower compared to the positive control.

Significant differences in NO levels were demonstrated in cultures pre-incubated with THP-1 supernatant (*F*_25_ = 15.999; *p* < 0.001). In these cultures, TP1 (*p* = 0.001 for 10 µM; *p* < 0.001 for 50 µM and 100 µM), TP4 (*p* = 0.003 for 10 µM; *p* < 0.001 for 50 µM and 100 µM), TP5 (*p* < 0.001), and TP8-TP10 compounds (*p* < 0.001) at each tested concentration decreased the level of nitric oxide statistically significantly compared to the negative control (Fig. 4C). Nitric oxide level was also reduced for 50 µM (*p* = 0.001) and 100 µM (*p* < 0.001) TP6 and 100 µM TP7 (*p* < 0.001). Moreover, the reduction of NO level at a concentration of 100 µM of TP1 (*p* = 0.001), TP4 (*p* = 0.02), TP5 (*p* = 0.01), and TP10 (*p* = 0.003), and 50 µM and 100 µM of TP8 (*p* = 0.04 for 50 µM; *p* = 0.001 for 100 µM) was statistically significant even compared to the positive control. The NO level was significantly higher than in the positive control only at 10 µM concentrations of TP6 (*p* = 0.04) and TP7 (*p* = 0.008).

### Effect of tested compounds on DNA damage in tested conditions

**Fig. 5:**
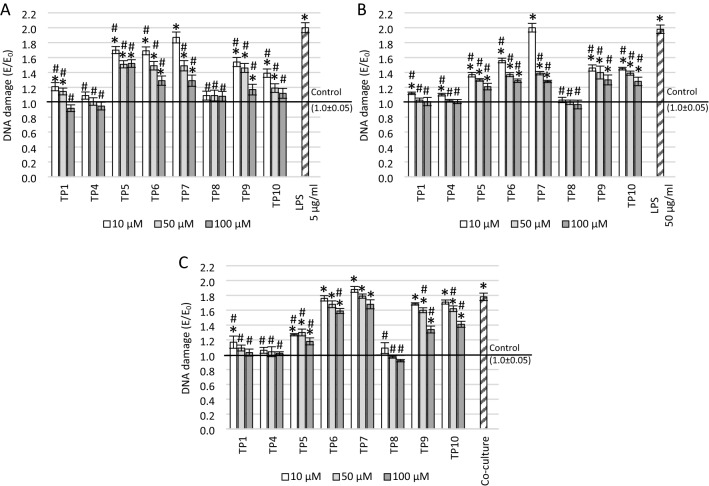
The effect of tested compounds on DNA damage measured in a fast halo assay (FHA). The SH-SY5Y neuronal cells were preincubated with: **A** 5 μg/ml lipopolysaccharide (LPS), **B** 50 μg/ml LPS, **c** supernatant from THP-1 cell culture; *p ≤ 0.05—significant difference compared to positive control without tested compounds, LPS or supernatant; ^#^*p* ≤ 0.05—significant difference compared to negative control with a harmful agent and without tested compounds. Presented data are mean ± SD of *n* = 5 independent experiments and were analyzed by one-way ANOVA with Tukey’s post hoc test

Since the level of ROS and NO can cause damage to the DNA strand, the FHA assay was also performed, which allows assessing the damage level. Sample micrographs with the nuclear halos are presented in Supplementary Materials. The one-way ANOVA showed statistical significance between groups treated with 5 µg/ml LPS (*F*_25_ = 119.67, *p* < 0.001), 50 µg/ml LPS (*F*_25_ = 187.59, *p* < 0.001), or supernatant from THP-1 cells (*F*_25_ = 469.93, *p* < 0.001). This study showed that incubation with LPS and the THP-1 supernatant caused DNA strand damages. All compounds tested induced concentration-dependent regeneration of strand breaks—the higher the concentration, the higher degree of regeneration (Fig. 5).

In cultures preincubated with 5 µg/ml and 50 µg/ml LPS, all compounds in each tested concentration (except for TP7 at the concentration of 10 µM—in this case, p = *NS)* reduced the number of DNA strand breaks compared to the LPS-treated negative control (*p* < 0.001; Fig. 5A, B). After preincubation with 5 µg/ml LPS, the reduction of the DNA strand breaks to the level close to positive control was observed after the use of TP4 and TP8 in the entire concentration range and TP1 and TP10 at the concentration of 100 µM. Tukey's post-hoc analysis showed that statistically significant higher DNA damage compared to the positive control in cells preincubated with 5 µg/ml LPS was after the use of concentrations of 10 µM and 50 µM of TP1 (*p* < 0.001, *p* = 0.04, respectively), TP10 (*p* < 0.001) and each concentration of TP5 (*p* < 0.001), TP6 (*p* < 0.001), TP7 (*p* < 0.001), and TP9 (*p* < 0.001 for 10 µM and 50 µM; *p* = 0.004 for 100 µM). In cultures preincubated with 50 µg/ml LPS, the DNA damage level was similar to the positive control for TP8 at each concentration and TP1 and TP4 at 50 µM and 100 µM. Tukey's post-hoc analysis showed that statistically significant higher DNA damage compared to the positive control was found after the use of 10 µM concentrations of TP1 (*p* = 0.003), TP4 (*p* = 0.03), and each concentration of TP5, TP6, TP7, TP9, and TP10 (*p* < 0.001).

Statistically significant reduction in the number of DNA strand breaks after preincubation with the THP-1 supernatant was observed in the entire concentration range of TP1, TP4, TP5, and TP8 compounds (*p* < 0.001), TP9 and TP10 compounds at a concentration of 50 µM (*p* < 0.001) and 100 µM (*p* < 0.001), and 100 µM TP6 (*p* < 0.001; Fig. 5C). Reduction of DNA strand breaks to the level close to positive control was observed in cultures with TP1 (except for 10 µM), TP4, and TP8 compounds. Tukey’s post-hoc analysis showed that statistically significant higher DNA damage compared to the positive control was observed after the use of all concentrations of TP5, TP6, TP7, TP9, and TP10 compounds (*p* < 0.001) and 10 µM TP1 (*p* < 0.001).

### Cyclooxygenase (COX) activity

**Fig. 6:**
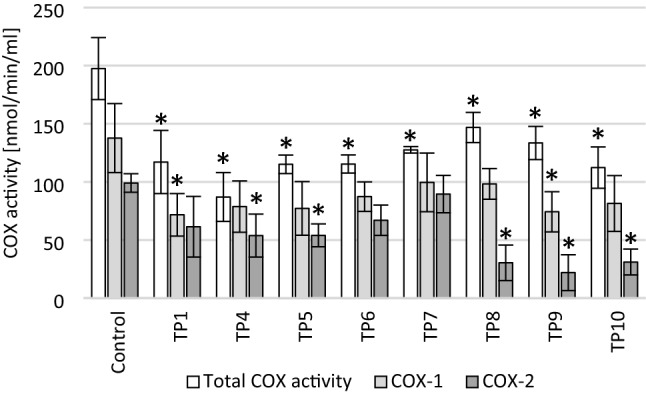
The effect of tested compounds at a concentration of 100 µM on cyclooxygenase (COX) activity. The SH-SY5Y neuronal cells were preincubated with 50 μg/ml LPS for 24 h; *p ≤ 0.05—a significant difference compared to the control without tested compounds. Presented data are mean ± SD of *n* = 3 independent experiments and were analyzed by one-way ANOVA with Tukey’s post hoc test

Additionally, for cultures preincubated with 50 µg/ml LPS, the COX-dependent peroxidative activity was assessed to determine if tested compounds affected the COX activity. One-way ANOVA analysis showed a statistically significant effect on the total COX level (*F*_8_ = 9.461282, *p* < 0.001), COX-1 level (*F*_8_ = 2.803689, *p* = 0.03) and COX-2 level (*F*_8_ = 8.521548, *p* < 0.001). Incubation with each tested compound reduced the total COX activity—for compounds: TP1, TP4, TP5, TP6, TP10 (*p* < 0.001), TP7 (*p* = 0.003), TP8 (*p* = 0.04) and TP9 (*p* = 0.006; Fig. 6).

Analysis of the effect on the COX-1 activity showed significant inhibition only for TP1 (*p* = 0.028) and TP9 (*p* = 0.037) compounds, which means that the remaining compounds did not significantly reduce the activity of COX-1. This can be considered a favorable property, as inhibition of COX-1 is responsible for the side effects of COX inhibitors.

Significant COX-2 inhibition was observed for most of the compounds tested, with the exception of TP1, TP6, and TP7. Among them, the compounds that decreased COX-2 activity most strongly were TP8 (*p* = 0.001), TP9 (*p* < 0.001) and TP10 (*p* = 0.001), and also showed the highest selectivity toward COX-2. A statistically significant reduction in COX-2 activity was also observed for the compounds TP4 (*p* = 0.047) and TP5 (*p* = 0.048).

### Computational studies on BBB permeation

The meaningful feature of drugs is their ability to penetrate the blood–brain barrier (BBB). It is important not only for the central nervous system (CNS) potential drugs but also for non-CNS therapies to minimize the unwanted CNS side effects [30]. However, measuring blood–brain barrier penetration is usually difficult and generates high costs. Therefore, a good alternative in silico prediction of BBB permeability is the early stage of drug design. The measure of the blood–brain barrier penetration is logBB = log(*C*_brain_/*C*_blood_), where *C*_brain_ and *C*_blood_ are the concentrations of the drug in the brain and the blood, respectively. Based on various combinations of physicochemical parameters using QSAR analysis, many models have been proposed to determine logBB. Four of them were used to calculate the logBB of the studied compounds.

The lipophilicity of a molecule is an important factor influencing its BBB passage. In the first model, the correlation between logBB and logarithm of the octanol/water partition coefficient (log *P*) and polar surface area (PSA) was used (Eq. 1 [31]). The second one thermodynamic parameter-free energy of solvation *G*_solv_ was calculated Eq. 2 [32]. Models 3 and 4 represent linear free-energy relationship (LFER), suggested by Abraham [33], with molecular descriptors: hydrogen-bond acidity (*A*) and basicity (*B*), polarizability (*S*), molar refraction (*E*), and the McGowan volume (*V*) of a solute (Eqs. 3 [33], 4 [34]).

1 $$\log {\text{BB}} = 0.139 + 0.152\log P - 0.0148PSA$$

2 $$\log {\text{BB}} = 0.054G_{w} + 0.43$$

3 $$\log {\text{BB}} = 0.044 + 0.511E - 0.886S - 0.724A - 0.666B + 0.861V$$

4 $$\log {\text{BB}} = 0.934 - 0.743A - 0.768B - 0.605 + 0.191E + 0.545V.$$

It implies the molecules with logBB > 0.3 cross the blood–brain barrier readily, while molecules with logBB < −1 are poorly distributed to the brain [35, 36]. The calculated values of the blood–brain partition coefficient are listed in Table 2. LogBB obtained from Eqs. 1 and 2 is below zero but above − 1. Newer models (Eqs. 3, 4) show slightly positive values (except TP1). These results indicate a good ability of studied compounds to penetrate the blood–brain barrier, and they will very likely be able to be active in the central nervous system [36]. However, the compound TP1 seems to be the worst candidate. We also calculated the probability of crossing the blood–brain by a procedure using machine learning and resampling methods [37]. For all compounds, the obtained probability is 0.95–0.97 (Table 2).

**Table 2:** Blood–brain partition coefficient (logBB) obtained from Eqs. 1–4 and probability of crossing the blood–brain barrier (AdmetSAR web tool)

	logBB				BBB crossing probability
	Equation 1	Equation 2	Equation 3	Equation 4	
TP1	− 0.94	− 0.26	− 1.11	− 0.99	0.9614
TP4	− 0.54	− 0.23	− 0.03	0.11	0.9738
TP5	− 0.47	− 0.27	0.09	0.19	0.9735
TP6	− 0.33	− 0.28	0.23	0.24	0.9735
TP7	− 0.37	− 0.27	0.15	0.21	0.9735
TP8	− 0.38	− 0.27	0.25	0.29	0.9733
TP9	− 0.59	− 0.32	0.00	0.05	0.9733
TP10	− 0.39	− 0.21	0.22	0.22	0.9742

### Prediction of ADMET properties

The ability of potential AD drugs to cross the blood–brain barrier is an important factor. However, it is also useful to predict ADMET (Absorption, Distribution, Metabolism, Excretion and Toxicity) parameters when designing new drugs. For accurate and comprehensive predictions of ADMET properties, the ADMETlab2.0 online platform was used [38]. Selected parameters are shown in Table 3 (all calculated parameters are listed in Supplementary Materials).

**Table 3:** ADMET properties of TP1–TP10

Parameter	Compound							
	TP1	TP4	TP5	TP6	TP7	TP8	TP9	TP10
*Absorption*								
Caco-2 Permeability	− 4.475	− 4.574	− 4.637	− 4.663	− 4.650	− 4.695	− 4.660	− 4.701
MDCK Permeability	2.71 × 10^–5^	3.23 × 10^–5^	2.67 × 10^–5^	2.49 × 10^–5^	2.09 × 10^–5^	2.53 × 10^–5^	2.78 × 10^–5^	2.50 × 10^–5^
*Distribution*								
VD	0.436	0.517	0.559	0.608	0.583	0.550	0.498	0.570
BBB Penetration	Yes	Yes	Yes	Yes	Yes	Yes	Yes	Yes
*Metabolism*								
CYP1A2	0.657	0.951	0.955	0.924	0.934	0.887	0.901	0.947
CYP2C19	0.539	0.977	0.977	0.968	0.974	0.969	0.973	0.975
CYP2C9	0.659	0.955	0.961	0.965	0.965	0.961	0.963	0.963
CYP2D6	0.068	0.586	0.602	0.611	0.700	0.607	0.563	0.567
CYP3A4	0.687	0.766	0.850	0.815	0.821	0.897	0.923	0.840
*Excretion*								
*T*_1/2_	0.148	0.313	0.220	0.089	0.095	0.130	0.162	0.211
*Toxicity*								
hERG Blockers	0.013	0.027	0.035	0.052	0.106	0.057	0.127	0.041
AMES Toxicity	0.046	0.236	0.114	0.133	0.159	0.182	0.272	0.097

Caco-2—log cm/s (proper value > –5.15log cm/s), MDCK—cm/s (high > 2 × 10^–5^ cm/s, low < 0.2 × 10^–5^ cm/s), CYP, *T*_1/2_, hERG, AMES—probability, VD—L/kg (proper range 0.04–20 L/kg)

For all compounds, a Caco-2 permeability value (colon adenocarcinoma cell lines), is above − 5.15 log cm/s. It’s indicated high intestinal absorption. The second absorption factor, Madin–Darby Canine Kidney cells, showed high passive MDCK permeability for all compounds. The VD parameter relates the administered drug dose with the actual initial concentration. It is predicted the compounds have proper VD values in the range of 0.04–20 L/kg. The prediction results indicate that compounds TP4–TP10 have high values for the probabilities of inhibiting human cytochrome isomers CYP1A2, CYP2C19, CYP2C9, CYP3A4 and moderate for CYP2D6. The human ether-a-go-go related gene (hERG) plays a major role in the passage of potassium ions through the cellular membrane. The probability that the tested compounds will block the hERG is very low. AMES toxicity is a mutagenicity test that has a close relationship with carcinogenicity. The obtained results indicate that the studied compounds should be non-toxic.

### Molecular docking with TLR4/MD-2 complex

**Fig. 7:**
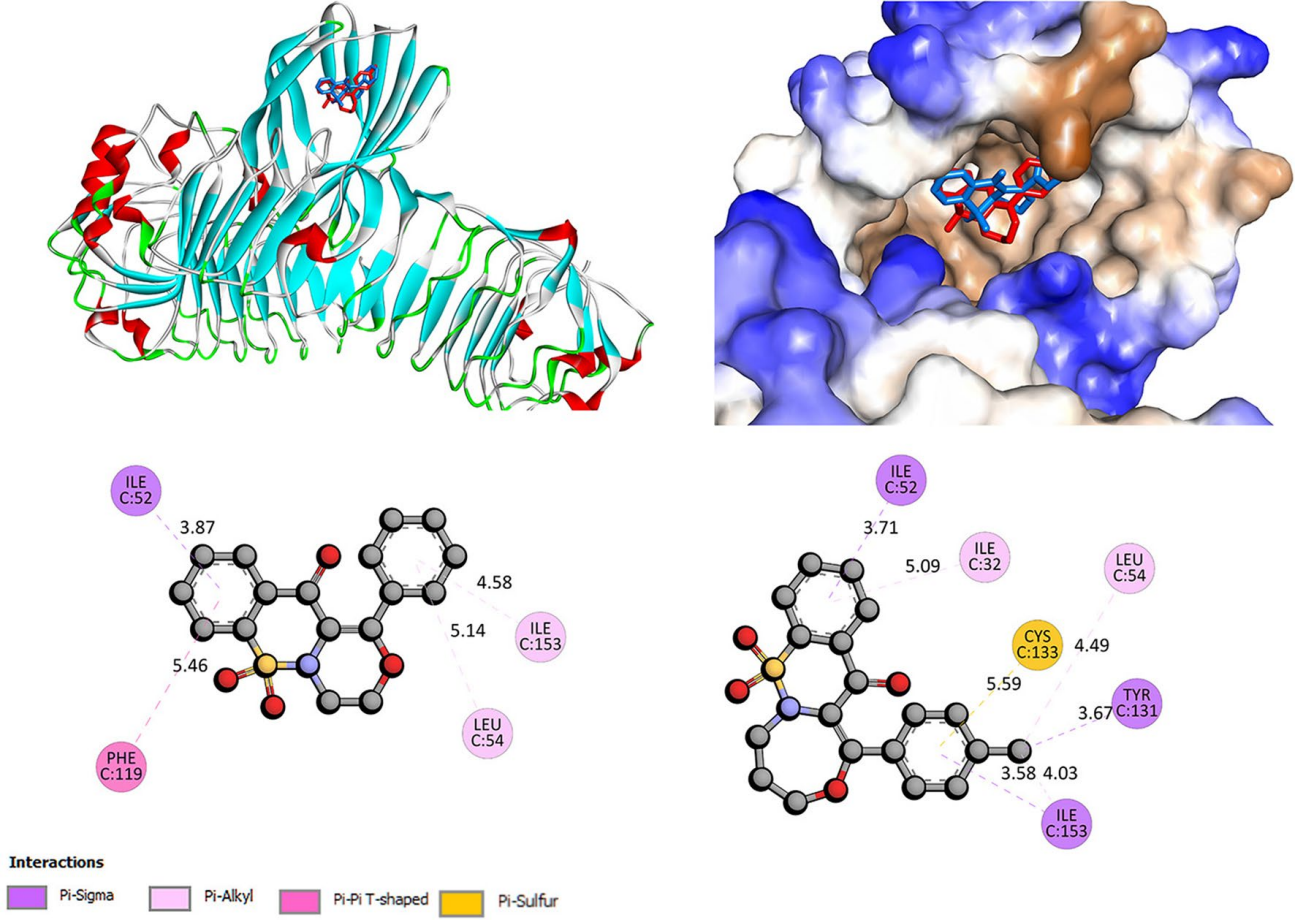
The docked pose of TP4 (blue) and TP8 (red) into the pocket site of TLR4/MD-2 complex and 2D interaction plot (TP4—left, TP8—right)

Toll-like receptors (TLRs) play an important role in inflammatory, autoimmune, and neurodegenerative disorders, including Alzheimer’s disease [39–42]. The TLR4/MD-2 complex interacts with LPS [43, 44], allowing the induction of a downstream signaling cascade, which may contribute to the worsening of neurodegenerative diseases [39, 42]. The studied compounds were docked at the LPS binding pocket. For all, the stable complex with TLR4/MD-2 is formed with binding affinity in the range from − 8.1 to − 9.7 kcal/mol for the best poses. Figure 7 shows the interactions and position of binding site compounds TP4 and TP 8, two molecules with the most promising neuroregenerative effect. The binding affinity was found as − 9.2 kcal/mol for TP4 and − 8.9 kcal/mol for TP8. The reference value for Donepezil, a drug used in AD treatment [45, 46], is reported as − 9.14 kcal/mol [47] (AutoDock scoring function, our studies − 9.1 kcal/mol, AutoDock Vina scoring function). It was reported that Leu54, Lys89, Arg90, Lys91, Lys122, Ile124, Lys125, Lys128, Tyr131 and Lys132 are the essential site for the LPS to bind for the microglia to be activated [43, 44, 47]. The molecular docking studies showed that TP4 and TP8 can bind in the same region. Hydrogen bonds are not observed. Leu 54 residue with phenyl ring of TP4 via π-alkyl contact and with –CH_3_ substituent of a phenyl ring of TP8 via π-sigma contact. Tyr131 also interacts via π-sigma contact with -CH_3_ group of the phenyl ring. Between the phenyl ring of TP4, TP8, and Ile153 hydrophobic interaction is formed. For TP8 π-sulfur, contact with Cys133 is observed. 1,2-benzothiazine moiety of TP4 binds to Ile52, Phe119 and to Ile52 and Ile32 for TP8. Details of interactions are presented in Fig. 7. Molecular docking results suggest that compounds TP4 and TP8 can cause a disturbance of LPS to interact with the amino acids located at the binding site of the TLR4/MD-2 complex. Other tested compounds interact similarly with TLR4/MD-2 complex. The details of the interactions are presented in the Supplementary Materials.

### Multiple-criteria decision analysis

The obtained results of all tests performed for newly synthesized eight derivatives at a concentration of 10 µM after initial incubation with 50 µg/ml LPS were subjected to multiple-criteria decision analysis (MCDA).

**Fig. 8:**
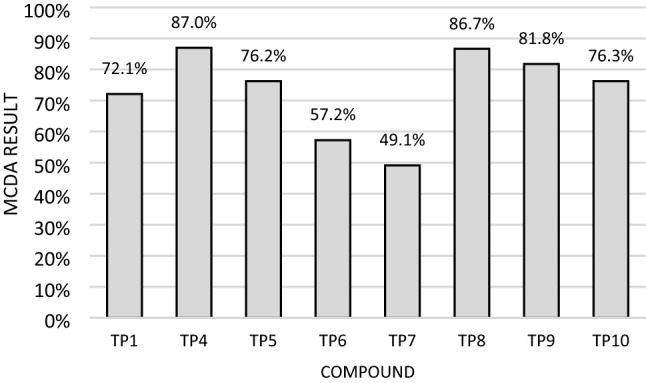
Multiple-criteria decision analysis (MCDA) of the effect of the tested compounds at a concentration of 10 µM in cultures preincubated with 50 µg/ml lipopolysaccharide (LPS); MCDA was calculated using the weighted sum model based on *E*/*E*_LPS50_ ratios determined in individual assays, where *E* is the sample result, and *E*_LPS50_ is the result in control preincubated with LPS at a concentration of 50 µg/ml

The MCDA results (Fig. 8) showed that compounds TP4 and TP8 had the strongest beneficial neuroregenerative effect on SH-SY5Y cells, with 87.0% and 86.7% values, respectively. In addition, the compound TP9 showed similar strong activity to them (81.8%). TP1, TP5, and TP10 compounds turned out to be not much weaker (72.1%, 76.2% and 76.3%, respectively). The weakest activity was obtained for TP6 and TP7 compounds (57.2% and 49.1%).

## Discussion

Alzheimer’s disease (AD) is a complex and multifactorial chronic neurodegenerative disease leading to progressive dementia that manifests as the loss of memory and language followed by disorientation and hallucinations. Currently, almost fifty million people live with dementia and AD is considered the most common cause of dementia among older people. However, there is still no effective treatment, and therefore studies on new drugs that might influence the course and progression of the disease are urgently needed. Since AD is an incurable, progressive disease, patients with AD require constant care and significant expenditures for therapy. Preventive strategies that can help delay the onset of AD and treatments that would slow down the progression of dementia will significantly help in reducing the global economic burden and societal impact of this disease. Although old age is the primary risk factor for AD, many other risks and protective factors may affect the progression or development of AD. They can be grouped into two main domains: non-modifiable and modifiable risk factors. Non-modifiable risk factors include age and genetic factors, and age seems to be the strongest and most important risk factor of AD [48]. Among modifiable ones are, among others, obesity, vascular disease, neurotoxins, chronic inflammation and social background. Activated microglia induces neuroinflammation that leads to neuronal damage and cognitive dysfunction. Modulating those risk factors and targeting the immune mechanisms involved in AD pathogenesis could lead to new future therapeutic or preventive strategies for Alzheimer’s disease. Regulation of neuroinflammation may become a potential strategy to alleviate the associated cognitive decline.

In the reported study, we investigated the potential influence of new tricyclic 1,2-thiazine derivatives on neuroinflammation and their ability to cross the blood–brain barrier. The main conclusion of our study is that compounds tested 1,2-thiazine derivatives could exert a neuroregenerative effect in inflammatory conditions. Additionally, in silico studies suggest that they would cross the blood–brain barrier.

The tested compounds generally did not decrease the viability of SH-SY5Y cells in the tested concentration. The only exceptions were the highest concentrations (100 µM) of TP8 and TP9 that reduced cell viability, which may be due to the presence of electron-donating groups (CH_3_ or OCH_3_) in their structure. However, lower concentrations of both compounds revealed no cytotoxic effect. What is more, TP1 in all tested concentrations and TP4 and TP7 in the lowest concentration even increased cell proliferation. Additionally, investigated compounds did not induce ROS and NO production and exerted no genotoxic effect in SH-SY5Y cells. Similar effects were reported for NHDFs (Normal Human Dermal Fibroblasts) [25]. Compound TP1 (4a in [25]) in all concentrations and TP7 (6c in [25]) in the lowest one increased the viability of NHDFs and tested compounds did not increase NO and ROS production in NHDFs.

LPS in both tested concentrations (5 µg/ml and 50 µg/ml) and co-culture with THP-1 cells, the previously described model of microglia activation [49], decreased the viability of SH-SY5Y cells, increased ROS and NO production, and induced double-strand DNA damage. Those results are in line with other authors reporting the injurious influence of inflammation on neuron-like cells [50, 51]. Potyrak et al. reported a similar influence of preincubation with LPS or co-culture with TPH-1 on ROS and NO production and DNA damage in SH-SY5Y cells [52].

In our study, the unfavorable effect of LPS and co-culture with TPH-1 cells on neuronal cell viability was counteracted with TP1 and TP4 in all tested concentrations. The beneficial properties of these compounds were certainly influenced by their chemical structure. Compound TP1 is structurally different from the rest of the compounds tested in that it is a bicyclic 1,2-benzothiazine derivative, while the compound TP4 is the only tricyclic derivative with a six-membered third ring. This may suggest that compounds with spatially smaller structures have better neuroregenerative activity.

Regardless of the type of preincubation, a significant effect was also noted in the lowest concentration of TP5, TP7, and TP8 compounds. In some cases, a positive effect was also shown for TP6 and TP9 at the concentration of 10 µM.

TP10 did not improve neuronal viability in inflammatory conditions at all, which may result from its different structure, as it is the only tricyclic compound with the eight-membered ring of oxazocin in its structure. This may suggest a significant influence of the size of the third ring on the neuroregenerative properties of the compound and that an eight-membered ring is unfavorable.

The investigated components also reduced the ROS level. However, TP6 and TP7 were the least effective, which may result from the presence of electron-withdrawing groups (Br or Cl) in their structure. This may suggest a significant influence of the substituent on the free radical scavenging properties of 1,2-benzothiazine tricyclic derivatives.

In the case of NO level, the compounds showed similar inhibitory properties as oxygen radicals. These findings suggest that the investigated compounds reduce the oxidative and nitrosative stress induced by both LPS and microglia activation. Oxidative stress plays an important role in the pathogenesis of AD, as the brain is more vulnerable to oxidative stress than other organs, and most of the neuronal structures can be oxidized. Furthermore, oxidative stress may induce amyloid plaques formation and *tau* hyperphosphorylation, which lead to progressive loss of synapses and neuronal damage [53]. Therefore, reducing ROS production by tested compounds is very important in light of their potential usage in AD therapy.

In our study, reduction in ROS and NO levels was accompanied by reduced DNA damage, except for the incubation with TP6 and TP7 compounds, which shows once again that electron-withdrawing substituents (Br, Cl) are not preferred in this case.

The MCDA, based on the results obtained for all tested compounds at the lowest tested concentration of 10 µM (in which the compounds showed the strongest activity) after initial incubation with 50 µg/ml LPS, allowed to identify of TP4, TP8 and TP9 as the compounds with potentially beneficial neuroregenerative effect on SH-SY5Y cells. Additionally, in LPS-stimulated SH-SY5Y cells, all tested compounds inhibited total COX activity and, in most cases, also COX-2. This observation is in line with results reported by Maniewska et al., that found that all investigated compounds inhibited COX-1 and COX-2 activity in the COX Colorimetric Inhibitor Screening Assay [25]. Maniewska et al. reported that they inhibited COX comparably to meloxicam [25]. The in vitro influence of investigated 1,2-thiazine derivatives on neuroinflammation was comparable to the previously reported influence of pyrrolo[3,4-*d*]pyridazinone derivatives [50, 52]. As both groups of components were reported to inhibit COX-1 and COX-2 activity [25, 54, 55], their similar neuroregenerative effect may be at least partially attributed to their anti-inflammatory activity associated with their influence on COX. As in the early stages of AD the overexpression of COX-2 has been reported [56, 57], the substances that inhibit COX activity may exert a protective effect against AD development.

An important aspect of the development of new drugs against AD is their permeability through the blood–brain barrier (BBB). The in silico analysis showed that the investigated compounds would cross the blood–brain barrier with high probability and that most of them (except TP1) would achieve in the brain the concentration not lower than in serum with TP6, TP7, TP8, and TP10 reaching the highest concentration in the brain.

The results of the in silico study of BBB permeability combined with the MCDA analysis indicating TP4, TP8 and TP9 as the compounds with the best neuroregenerative properties suggest that they are the best candidates for further research on their influence on AD. These three compounds are tricyclic 1,2-benzothiazine derivatives and differ in the type of the third ring. Compound TP4 is a derivative of a six-membered oxazine, while compounds TP8 and TP9 are derivatives of a seven-membered oxazepin with an additional methyl or methoxy substituent. This may suggest that six- and seven-membered rings are preferred for the neuroregenerative effect of this group of compounds rather than eight-membered rings, as in the case of TP10. Additionally, electron-donating substituents (CH_3_ and OCH_3_) seem to have also a beneficial effect in this group of compounds. As a pro-inflammatory response to LPS is attributed to the activation of TLR4 followed by the inflammatory cascade involving, among others, NF-κB and MAPK [58], it would be of great value to investigate the influence of TP4, TP8 and TP9 on those signaling pathways in the future.

Study limitations: our current study is an in silico and in vitro study. Human neuroblastoma cells SH-SY5Y were used in biological experiments. The limitation of this cell line is that it is cancerous and not nervous. These cells are commonly used as a model line for neurobiological research after appropriate preparation. Under the influence of a differentiating medium, it exhibits features characteristic of nerve cells. This line is often used in screening tests to evaluate newly synthesized compounds and elucidate molecular mechanisms before the isolation of primordial lines and in vivo experiments. Our study evaluates and selects the most promising newly synthesized compounds for counteracting the neuroinflammatory processes characteristic of AD. Computer-based ADMET analysis was performed to assess the pharmacokinetics of the tested compounds and the possibility of permeability of the blood–brain barrier. Undoubtedly, in silico experiments also have their limitations, e.g., in the ADMET evaluation, there is no assessment of liver metabolism and the functional blood–brain barrier. It should therefore be emphasized that this study does not cover purely pharmacokinetic or pharmacological aspects that could alter the potency of the effect in vivo. However, this study allows the selection of TP4 and TP8 compounds for further studies on primary neuronal cells and in vivo studies, thus limiting the number of animals necessary for experimental studies. Our results emphasize that the tested compounds block the LPS binding into TLR4/MD-2 and reduce the neuroinflammation effect induced by preincubation with LPS, reducing DNA double-strand breaks, as well as ROS and NO levels. Of course, further in vivo studies are needed to clarify whether the molecular mechanisms found here also lead to comparable effects concerning AD in humans.

## Conclusions

We demonstrated that new tricyclic 1,2-thiazine derivatives TP4, TP8 and TP9 could exert a neuroregenerative effect in the neuroinflammation model of neuronal damage and would probably cross the BBB reaching in the brain the concentration not lower than in the serum. Based on the ADMET analysis, it can be assumed that all tested compounds are characterized by good intestinal absorption and should be non-toxic. Moreover, in molecular docking, the tested compounds have been shown to bind to the TLR4/MD-2 complex, which plays an important role in Alzheimer's disease. The mechanism of the neuroregenerative activity of tested compounds could be attributed to their inhibitory influence on COX activity and reduction of oxidative and nitrosative stress. Those compounds could become a part of the future strategy of AD therapy. However, they require further investigation.

## Supplementary Information

Below is the link to the electronic supplementary material.

Supplementary file1 (DOCX 69 kb)

Supplementary file2 (PDF 376 kb)

Supplementary file3 (XLSX 31 kb)

Supplementary file4 (DOCX 718 kb)

## Data Availability

The data generated and analyzed during the current study are available from the corresponding author upon reasonable request.

## Abbreviations

AD: Alzheimer’s disease
ADMET: Absorption, Distribution, Metabolism, Excretion and Toxicity
AMPs: Antimicrobial peptides
APP: Amyloid protein precursor
BBB: Blood-brain barrier
CNS: Central nervous system
COX: Cyclooxygenase
DMSO: Dimethyl sulfoxide
FBS: Fetal bovine serum
FHA: Fast halo assay
LPS: Lipopolysaccharide
MEM: Modified Eagle Medium
NHDF: Normal Human Dermal Fibroblasts
PMA: Phorbol-12-myristate-13-acetate
RNS: Reactive nitrogen species
ROS: Reactive oxygen species
TMPPD: *N*,*N*,*N*′,*N*′-tetramethyl-p-phenylenediamine
