# Supplementary material for: Effect of tricyclic 1,2-thiazine derivatives in neuroinflammation induced by preincubation with lipopolysaccharide or coculturing with microglia-like cells
Source: Pharmacol Rep. 2022 Sep 21;74(5):890–908. doi: 10.1007/s43440-022-00414-8 (PMC9584986; doi:10.1007/s43440-022-00414-8)
Supplement: Supplementary file 1 — Supplementary file1 (DOCX 69 kb) [file 43440_2022_414_MOESM1_ESM.docx]

# MTT assay results

Table S1. MTT test results in SH-SY5Y cell culture

| Compounds | Concentration | Min | Max | Mean | SD |
| --- | --- | --- | --- | --- | --- |
| Positive Control | - | 0.97 | 1.05 | 1.00 | 0.03 |
| TP1 | 10 μM | 1.66 | 1.75 | 1.71 | 0.04 |
|  | 50 μM | 1.39 | 1.56 | 1.47 | 0.07 |
|  | 100 μM | 1.19 | 1.35 | 1.25 | 0.06 |
| TP4 | 10 μM | 1.40 | 1.62 | 1.50 | 0.11 |
|  | 50 μM | 0.85 | 1.04 | 0.97 | 0.07 |
|  | 100 μM | 0.81 | 1.02 | 0.89 | 0.08 |
| TP5 | 10 μM | 0.91 | 1.09 | 1.03 | 0.07 |
|  | 50 μM | 0.83 | 1.04 | 0.93 | 0.08 |
|  | 100 μM | 0.73 | 0.93 | 0.84 | 0.09 |
| TP6 | 10 μM | 0.98 | 1.22 | 1.09 | 0.10 |
|  | 50 μM | 0.99 | 1.08 | 1.04 | 0.04 |
|  | 100 μM | 0.91 | 1.09 | 0.99 | 0.07 |
| TP7 | 10 μM | 1.08 | 1.30 | 1.20 | 0.09 |
|  | 50 μM | 0.91 | 1.15 | 1.07 | 0.10 |
|  | 100 μM | 0.86 | 1.08 | 0.97 | 0.09 |
| TP8 | 10 μM | 0.90 | 1.15 | 1.04 | 0.11 |
|  | 50 μM | 0.79 | 0.97 | 0.91 | 0.07 |
|  | 100 μM | 0.67 | 0.83 | 0.74 | 0.06 |
| TP9 | 10 μM | 0.94 | 1.13 | 1.04 | 0.07 |
|  | 50 μM | 0.75 | 0.95 | 0.85 | 0.08 |
|  | 100 μM | 0.63 | 0.79 | 0.72 | 0.06 |
| TP10 | 10 μM | 0.84 | 1.06 | 0.96 | 0.08 |
|  | 50 μM | 0.76 | 0.93 | 0.83 | 0.08 |
|  | 100 μM | 0.76 | 0.92 | 0.85 | 0.07 |

Table S2. MTT test results in SH-SY5Y cell culture pre-incubated with 5 μg/ml LPS.

| Compounds | Concentration | Min | Max | Mean | SD |
| --- | --- | --- | --- | --- | --- |
| Positive Control | - | 0.97 | 1.05 | 1.00 | 0.03 |
| Negative control | 5 μg/ml LPS | 0.77 | 0.86 | 0.82 | 0.03 |
| TP1 | 10 μM | 1.24 | 1.37 | 1.30 | 0.04 |
|  | 50 μM | 1.26 | 1.31 | 1.28 | 0.02 |
|  | 100 μM | 1.05 | 1.19 | 1.10 | 0.05 |
| TP4 | 10 μM | 1.25 | 1.35 | 1.29 | 0.04 |
|  | 50 μM | 0.94 | 1.06 | 1.01 | 0.05 |
|  | 100 μM | 0.96 | 1.04 | 1.00 | 0.04 |
| TP5 | 10 μM | 0.89 | 1.10 | 0.99 | 0.09 |
|  | 50 μM | 0.84 | 1.08 | 0.92 | 0.11 |
|  | 100 μM | 0.70 | 1.00 | 0.88 | 0.11 |
| TP6 | 10 μM | 0.97 | 1.02 | 0.99 | 0.02 |
|  | 50 μM | 0.88 | 0.96 | 0.92 | 0.03 |
|  | 100 μM | 0.78 | 0.84 | 0.81 | 0.03 |
| TP7 | 10 μM | 1.07 | 1.30 | 1.18 | 0.09 |
|  | 50 μM | 0.96 | 1.07 | 1.03 | 0.04 |
|  | 100 μM | 0.83 | 1.02 | 0.94 | 0.08 |
| TP8 | 10 μM | 0.89 | 1.06 | 1.00 | 0.07 |
|  | 50 μM | 0.93 | 1.02 | 0.99 | 0.04 |
|  | 100 μM | 0.85 | 0.99 | 0.92 | 0.06 |
| TP9 | 10 μM | 0.86 | 0.95 | 0.90 | 0.04 |
|  | 50 μM | 0.73 | 0.96 | 0.84 | 0.11 |
|  | 100 μM | 0.59 | 0.92 | 0.73 | 0.12 |
| TP10 | 10 μM | 0.80 | 1.02 | 0.92 | 0.09 |
|  | 50 μM | 0.72 | 0.86 | 0.80 | 0.05 |
|  | 100 μM | 0.52 | 0.78 | 0.67 | 0.11 |

Table S3. MTT test results in SH-SY5Y cell culture pre-incubated with 50 μg/ml LPS.

| Compounds | Concentration | Min | Max | Mean | SD |
| --- | --- | --- | --- | --- | --- |
| Positive Control | - | 0.97 | 1.05 | 1.00 | 0.03 |
| Negative control | 50 μg/ml LPS | 0.55 | 0.68 | 0.61 | 0.05 |
| TP1 | 10 μM | 1.06 | 1.16 | 1.10 | 0.04 |
|  | 50 μM | 0.96 | 1.05 | 1.00 | 0.03 |
|  | 100 μM | 0.91 | 0.98 | 0.95 | 0.03 |
| TP4 | 10 μM | 0.96 | 1.22 | 1.09 | 0.10 |
|  | 50 μM | 0.89 | 1.08 | 0.98 | 0.07 |
|  | 100 μM | 0.73 | 0.98 | 0.89 | 0.11 |
| TP5 | 10 μM | 0.96 | 1.05 | 1.01 | 0.04 |
|  | 50 μM | 0.87 | 0.99 | 0.92 | 0.05 |
|  | 100 μM | 0.78 | 0.98 | 0.86 | 0.07 |
| TP6 | 10 μM | 0.71 | 0.86 | 0.81 | 0.06 |
|  | 50 μM | 0.67 | 0.83 | 0.75 | 0.06 |
|  | 100 μM | 0.53 | 0.72 | 0.65 | 0.08 |
| TP7 | 10 μM | 0.98 | 1.07 | 1.03 | 0.04 |
|  | 50 μM | 0.84 | 1.02 | 0.92 | 0.07 |
|  | 100 μM | 0.81 | 0.90 | 0.86 | 0.04 |
| TP8 | 10 μM | 1.02 | 1.13 | 1.08 | 0.04 |
|  | 50 μM | 0.90 | 1.07 | 0.98 | 0.07 |
|  | 100 μM | 0.88 | 0.94 | 0.92 | 0.03 |
| TP9 | 10 μM | 0.91 | 1.12 | 1.01 | 0.08 |
|  | 50 μM | 0.70 | 0.92 | 0.83 | 0.09 |
|  | 100 μM | 0.62 | 0.81 | 0.73 | 0.07 |
| TP10 | 10 μM | 0.67 | 0.75 | 0.70 | 0.03 |
|  | 50 μM | 0.63 | 0.73 | 0.67 | 0.04 |
|  | 100 μM | 0.56 | 0.65 | 0.60 | 0.04 |

Table S4. MTT test results in SH-SY5Y cell culture pre-incubated with supernatant from THP-1 cell culture.

| Compounds | Concentration | Min | Max | Mean | SD |
| --- | --- | --- | --- | --- | --- |
| Positive Control | - | 0.97 | 1.05 | 1.00 | 0.03 |
| Negative control | Co-culture | 0.64 | 0.77 | 0.70 | 0.05 |
| TP1 | 10 μM | 1.12 | 1.16 | 1.14 | 0.02 |
|  | 50 μM | 1.02 | 1.12 | 1.08 | 0.04 |
|  | 100 μM | 0.95 | 1.09 | 1.03 | 0.05 |
| TP4 | 10 μM | 1.09 | 1.16 | 1.13 | 0.03 |
|  | 50 μM | 0.97 | 1.06 | 1.02 | 0.03 |
|  | 100 μM | 0.87 | 0.97 | 0.92 | 0.05 |
| TP5 | 10 μM | 0.93 | 1.00 | 0.97 | 0.03 |
|  | 50 μM | 0.84 | 0.94 | 0.89 | 0.04 |
|  | 100 μM | 0.77 | 0.86 | 0.81 | 0.04 |
| TP6 | 10 μM | 0.78 | 0.86 | 0.82 | 0.03 |
|  | 50 μM | 0.76 | 0.83 | 0.80 | 0.03 |
|  | 100 μM | 0.74 | 0.85 | 0.79 | 0.04 |
| TP7 | 10 μM | 0.91 | 1.03 | 0.99 | 0.05 |
|  | 50 μM | 0.80 | 1.03 | 0.88 | 0.09 |
|  | 100 μM | 0.76 | 0.87 | 0.81 | 0.04 |
| TP8 | 10 μM | 0.89 | 1.17 | 1.04 | 0.10 |
|  | 50 μM | 0.91 | 0.98 | 0.95 | 0.03 |
|  | 100 μM | 0.78 | 0.89 | 0.82 | 0.04 |
| TP9 | 10 μM | 0.83 | 0.94 | 0.90 | 0.04 |
|  | 50 μM | 0.84 | 0.94 | 0.88 | 0.04 |
|  | 100 μM | 0.65 | 0.95 | 0.81 | 0.11 |
| TP10 | 10 μM | 0.63 | 0.87 | 0.77 | 0.09 |
|  | 50 μM | 0.69 | 0.79 | 0.73 | 0.04 |
|  | 100 μM | 0.66 | 0.79 | 0.71 | 0.05 |

# DCF-DA assay results

Table S5. MTT test results in SH-SY5Y cell culture

| Compounds | Concentration | Min | Max | Mean | SD |
| --- | --- | --- | --- | --- | --- |
| Positive Control | - | 0.95 | 1.06 | 1.00 | 0.04 |
| TP1 | 10μM | 0.99 | 1.06 | 1.02 | 0.03 |
|  | 50 μM | 0.99 | 1.06 | 1.03 | 0.03 |
|  | 100 μM | 0.96 | 1.07 | 1.02 | 0.04 |
| TP4 | 10μM | 0.98 | 1.05 | 1.02 | 0.03 |
|  | 50 μM | 0.97 | 1.07 | 1.02 | 0.04 |
|  | 100 μM | 0.98 | 1.04 | 1.01 | 0.02 |
| TP5 | 10μM | 0.98 | 1.03 | 1.01 | 0.02 |
|  | 50 μM | 0.96 | 1.07 | 1.01 | 0.04 |
|  | 100 μM | 0.97 | 1.04 | 1.01 | 0.03 |
| TP6 | 10μM | 0.97 | 1.08 | 1.04 | 0.04 |
|  | 50 μM | 0.99 | 1.06 | 1.03 | 0.03 |
|  | 100 μM | 0.97 | 1.04 | 1.01 | 0.03 |
| TP7 | 10μM | 0.96 | 1.01 | 0.99 | 0.02 |
|  | 50 μM | 1.01 | 1.03 | 1.02 | 0.01 |
|  | 100 μM | 1.00 | 1.04 | 1.02 | 0.01 |
| TP8 | 10μM | 0.97 | 1.06 | 1.01 | 0.03 |
|  | 50 μM | 0.98 | 1.03 | 1.00 | 0.02 |
|  | 100 μM | 0.97 | 1.04 | 1.00 | 0.03 |
| TP9 | 10μM | 0.98 | 1.01 | 1.00 | 0.01 |
|  | 50 μM | 0.98 | 1.02 | 1.00 | 0.02 |
|  | 100 μM | 0.98 | 1.02 | 1.00 | 0.02 |
| TP10 | 10μM | 0.99 | 1.01 | 1.00 | 0.01 |
|  | 50 μM | 0.97 | 1.02 | 1.00 | 0.02 |
|  | 100 μM | 0.96 | 1.01 | 0.98 | 0.02 |

Table S6. DCF-DA test results in SH-SY5Y cell culture pre-incubated with 5 μg/ml LPS.

| Compounds | Concentration | Min | Max | Mean | SD |
| --- | --- | --- | --- | --- | --- |
| Positive Control | - | 0.95 | 1.06 | 1.00 | 0.04 |
| Negative control | 5 μg/ml LPS | 1.26 | 1.54 | 1.40 | 0.10 |
| TP1 | 10μM | 1.03 | 1.18 | 1.08 | 0.06 |
|  | 50 μM | 0.95 | 1.10 | 1.03 | 0.06 |
|  | 100 μM | 0.84 | 0.99 | 0.91 | 0.05 |
| TP4 | 10μM | 0.98 | 1.10 | 1.04 | 0.05 |
|  | 50 μM | 1.00 | 1.13 | 1.05 | 0.05 |
|  | 100 μM | 0.85 | 0.98 | 0.94 | 0.05 |
| TP5 | 10μM | 1.02 | 1.16 | 1.08 | 0.05 |
|  | 50 μM | 0.95 | 1.09 | 1.03 | 0.05 |
|  | 100 μM | 0.90 | 1.04 | 0.95 | 0.05 |
| TP6 | 10μM | 1.25 | 1.39 | 1.31 | 0.05 |
|  | 50 μM | 1.18 | 1.32 | 1.25 | 0.06 |
|  | 100 μM | 1.03 | 1.18 | 1.11 | 0.06 |
| TP7 | 10μM | 1.18 | 1.37 | 1.25 | 0.07 |
|  | 50 μM | 1.09 | 1.26 | 1.17 | 0.07 |
|  | 100 μM | 0.99 | 1.19 | 1.07 | 0.07 |
| TP8 | 10μM | 0.88 | 1.03 | 0.97 | 0.06 |
|  | 50 μM | 0.87 | 1.06 | 0.93 | 0.07 |
|  | 100 μM | 0.83 | 1.00 | 0.92 | 0.07 |
| TP9 | 10μM | 0.91 | 1.07 | 0.98 | 0.06 |
|  | 50 μM | 0.84 | 1.00 | 0.94 | 0.06 |
|  | 100 μM | 0.83 | 0.99 | 0.92 | 0.07 |
| TP10 | 10μM | 0.93 | 1.08 | 1.00 | 0.06 |
|  | 50 μM | 0.86 | 1.04 | 0.94 | 0.06 |
|  | 100 μM | 0.87 | 1.03 | 0.92 | 0.07 |

Table S7. DCF-DA test results in SH-SY5Y cell culture pre-incubated with 50 μg/ml LPS.

| Compounds | Concentration | Min | Max | Mean | SD |
| --- | --- | --- | --- | --- | --- |
| Positive Control | - | 0.95 | 1.06 | 1.00 | 0.04 |
| Negative control | 50 μg/ml LPS | 1.87 | 1.94 | 1.90 | 0.03 |
| TP1 | 10μM | 1.14 | 1.33 | 1.24 | 0.07 |
|  | 50 μM | 1.23 | 1.27 | 1.25 | 0.02 |
|  | 100 μM | 1.01 | 1.07 | 1.04 | 0.02 |
| TP4 | 10μM | 1.16 | 1.24 | 1.20 | 0.03 |
|  | 50 μM | 1.04 | 1.20 | 1.12 | 0.06 |
|  | 100 μM | 1.00 | 1.07 | 1.04 | 0.03 |
| TP5 | 10μM | 1.24 | 1.33 | 1.29 | 0.03 |
|  | 50 μM | 1.04 | 1.18 | 1.12 | 0.05 |
|  | 100 μM | 0.99 | 1.11 | 1.04 | 0.04 |
| TP6 | 10μM | 1.49 | 1.62 | 1.54 | 0.05 |
|  | 50 μM | 1.27 | 1.45 | 1.35 | 0.08 |
|  | 100 μM | 1.15 | 1.29 | 1.21 | 0.07 |
| TP7 | 10μM | 1.34 | 1.50 | 1.42 | 0.06 |
|  | 50 μM | 1.24 | 1.35 | 1.30 | 0.04 |
|  | 100 μM | 1.16 | 1.23 | 1.19 | 0.03 |
| TP8 | 10μM | 1.02 | 1.15 | 1.07 | 0.05 |
|  | 50 μM | 0.96 | 1.01 | 0.99 | 0.02 |
|  | 100 μM | 0.86 | 1.04 | 0.95 | 0.08 |
| TP9 | 10μM | 1.14 | 1.18 | 1.16 | 0.02 |
|  | 50 μM | 0.98 | 1.06 | 1.02 | 0.03 |
|  | 100 μM | 0.82 | 1.07 | 0.95 | 0.10 |
| TP10 | 10μM | 0.97 | 1.10 | 1.03 | 0.05 |
|  | 50 μM | 0.89 | 1.00 | 0.94 | 0.04 |
|  | 100 μM | 0.87 | 0.98 | 0.94 | 0.04 |

Table S8. DCF-DA test results in SH-SY5Y cell culture pre-incubated with supernatant from THP-1 cell culture.

| Compounds | Concentration | Min | Max | Mean | SD |
| --- | --- | --- | --- | --- | --- |
| Positive Control | - | 0.95 | 1.06 | 1.00 | 0.04 |
| Negative control | Co-culture | 1.52 | 1.63 | 1.58 | 0.04 |
| TP1 | 10μM | 1.05 | 1.11 | 1.07 | 0.02 |
|  | 50 μM | 0.97 | 1.05 | 1.02 | 0.03 |
|  | 100 μM | 0.95 | 1.04 | 1.00 | 0.03 |
| TP4 | 10μM | 0.97 | 1.11 | 1.05 | 0.05 |
|  | 50 μM | 0.97 | 1.06 | 1.01 | 0.03 |
|  | 100 μM | 0.98 | 1.03 | 1.01 | 0.02 |
| TP5 | 10μM | 1.09 | 1.15 | 1.13 | 0.02 |
|  | 50 μM | 1.06 | 1.15 | 1.09 | 0.04 |
|  | 100 μM | 0.97 | 1.03 | 1.00 | 0.02 |
| TP6 | 10μM | 1.29 | 1.40 | 1.34 | 0.04 |
|  | 50 μM | 1.24 | 1.36 | 1.30 | 0.05 |
|  | 100 μM | 1.13 | 1.24 | 1.18 | 0.05 |
| TP7 | 10μM | 1.25 | 1.29 | 1.28 | 0.02 |
|  | 50 μM | 1.15 | 1.28 | 1.22 | 0.05 |
|  | 100 μM | 1.13 | 1.21 | 1.16 | 0.03 |
| TP8 | 10μM | 0.99 | 1.06 | 1.03 | 0.03 |
|  | 50 μM | 0.96 | 1.07 | 1.00 | 0.04 |
|  | 100 μM | 0.94 | 1.04 | 0.99 | 0.04 |
| TP9 | 10μM | 0.99 | 1.06 | 1.02 | 0.02 |
|  | 50 μM | 0.94 | 1.09 | 0.99 | 0.06 |
|  | 100 μM | 0.89 | 0.96 | 0.94 | 0.03 |
| TP10 | 10μM | 0.94 | 1.05 | 0.99 | 0.04 |
|  | 50 μM | 0.92 | 0.98 | 0.95 | 0.02 |
|  | 100 μM | 0.91 | 0.97 | 0.94 | 0.02 |

# Griess assay results

Table S9. Griess test results in SH-SY5Y cell culture

| Compounds | Concentration | Min | Max | Mean | SD |
| --- | --- | --- | --- | --- | --- |
| Positive Control | - | 0.93 | 1.03 | 1.00 | 0.04 |
| TP1 | 10μM | 0.82 | 0.95 | 0.90 | 0.05 |
|  | 50 μM | 0.84 | 0.96 | 0.88 | 0.05 |
|  | 100 μM | 0.80 | 0.93 | 0.86 | 0.05 |
| TP4 | 10μM | 0.82 | 0.96 | 0.89 | 0.06 |
|  | 50 μM | 0.85 | 0.94 | 0.89 | 0.05 |
|  | 100 μM | 0.84 | 0.96 | 0.91 | 0.05 |
| TP5 | 10μM | 0.81 | 0.96 | 0.91 | 0.06 |
|  | 50 μM | 0.84 | 0.99 | 0.93 | 0.06 |
|  | 100 μM | 0.84 | 0.95 | 0.89 | 0.05 |
| TP6 | 10μM | 0.83 | 0.96 | 0.91 | 0.05 |
|  | 50 μM | 0.83 | 0.93 | 0.88 | 0.05 |
|  | 100 μM | 0.82 | 1.00 | 0.89 | 0.07 |
| TP7 | 10μM | 0.81 | 0.95 | 0.88 | 0.06 |
|  | 50 μM | 0.79 | 0.95 | 0.87 | 0.07 |
|  | 100 μM | 0.83 | 0.98 | 0.88 | 0.06 |
| TP8 | 10μM | 0.80 | 0.95 | 0.87 | 0.06 |
|  | 50 μM | 0.79 | 0.93 | 0.86 | 0.06 |
|  | 100 μM | 0.77 | 0.92 | 0.87 | 0.06 |
| TP9 | 10μM | 0.81 | 0.95 | 0.87 | 0.06 |
|  | 50 μM | 0.80 | 0.96 | 0.88 | 0.06 |
|  | 100 μM | 0.84 | 0.97 | 0.89 | 0.06 |
| TP10 | 10μM | 0.83 | 0.96 | 0.90 | 0.06 |
|  | 50 μM | 0.80 | 0.93 | 0.88 | 0.06 |
|  | 100 μM | 0.80 | 0.96 | 0.90 | 0.06 |

Table S10. Griess test results in SH-SY5Y cell culture pre-incubated with 5 μg/ml LPS.

| Compounds | Concentration | Min | Max | Mean | SD |
| --- | --- | --- | --- | --- | --- |
| Positive Control | - | 0.93 | 1.03 | 1.00 | 0.04 |
| Negative control | 5 μg/ml LPS | 1.30 | 1.43 | 1.37 | 0.05 |
| TP1 | 10μM | 0.99 | 1.20 | 1.07 | 0.08 |
|  | 50 μM | 0.97 | 1.12 | 1.02 | 0.06 |
|  | 100 μM | 0.94 | 0.99 | 0.97 | 0.02 |
| TP4 | 10μM | 1.04 | 1.13 | 1.08 | 0.04 |
|  | 50 μM | 1.00 | 1.08 | 1.05 | 0.03 |
|  | 100 μM | 0.98 | 1.06 | 1.02 | 0.03 |
| TP5 | 10μM | 1.07 | 1.17 | 1.13 | 0.04 |
|  | 50 μM | 1.08 | 1.16 | 1.12 | 0.04 |
|  | 100 μM | 1.10 | 1.12 | 1.11 | 0.01 |
| TP6 | 10μM | 1.30 | 1.36 | 1.32 | 0.02 |
|  | 50 μM | 1.17 | 1.24 | 1.20 | 0.03 |
|  | 100 μM | 1.11 | 1.24 | 1.19 | 0.05 |
| TP7 | 10μM | 1.30 | 1.51 | 1.41 | 0.07 |
|  | 50 μM | 1.21 | 1.37 | 1.28 | 0.06 |
|  | 100 μM | 1.11 | 1.21 | 1.17 | 0.04 |
| TP8 | 10μM | 1.01 | 1.03 | 1.02 | 0.01 |
|  | 50 μM | 0.91 | 1.05 | 0.98 | 0.05 |
|  | 100 μM | 0.87 | 0.91 | 0.89 | 0.02 |
| TP9 | 10μM | 1.02 | 1.10 | 1.06 | 0.03 |
|  | 50 μM | 0.88 | 0.97 | 0.92 | 0.04 |
|  | 100 μM | 0.83 | 0.91 | 0.87 | 0.03 |
| TP10 | 10μM | 0.95 | 1.15 | 1.05 | 0.07 |
|  | 50 μM | 0.92 | 0.97 | 0.95 | 0.02 |
|  | 100 μM | 0.87 | 0.91 | 0.89 | 0.01 |

Table S11. Griess test results in SH-SY5Y cell culture pre-incubated with 50 μg/ml LPS.

| Compounds | Concentration | Min | Max | Mean | SD |
| --- | --- | --- | --- | --- | --- |
| Positive Control | - | 0.93 | 1.03 | 1.00 | 0.04 |
| Negative control | 50 μg/ml LPS | 1.66 | 1.83 | 1.72 | 0.07 |
| TP1 | 10μM | 0.95 | 1.08 | 1.02 | 0.06 |
|  | 50 μM | 0.90 | 1.08 | 0.98 | 0.08 |
|  | 100 μM | 0.92 | 0.97 | 0.95 | 0.02 |
| TP4 | 10μM | 1.02 | 1.10 | 1.07 | 0.03 |
|  | 50 μM | 0.96 | 1.06 | 1.02 | 0.04 |
|  | 100 μM | 0.91 | 1.05 | 0.99 | 0.05 |
| TP5 | 10μM | 1.08 | 1.12 | 1.10 | 0.02 |
|  | 50 μM | 0.92 | 1.15 | 1.03 | 0.09 |
|  | 100 μM | 0.91 | 1.08 | 0.99 | 0.06 |
| TP6 | 10μM | 1.26 | 1.33 | 1.29 | 0.03 |
|  | 50 μM | 1.21 | 1.25 | 1.23 | 0.02 |
|  | 100 μM | 1.05 | 1.18 | 1.10 | 0.06 |
| TP7 | 10μM | 1.41 | 1.54 | 1.49 | 0.05 |
|  | 50 μM | 1.34 | 1.42 | 1.38 | 0.03 |
|  | 100 μM | 1.13 | 1.36 | 1.26 | 0.10 |
| TP8 | 10μM | 0.94 | 1.04 | 0.97 | 0.04 |
|  | 50 μM | 0.83 | 0.89 | 0.86 | 0.03 |
|  | 100 μM | 0.80 | 0.88 | 0.85 | 0.03 |
| TP9 | 10μM | 0.95 | 1.09 | 1.03 | 0.05 |
|  | 50 μM | 0.84 | 0.98 | 0.92 | 0.05 |
|  | 100 μM | 0.77 | 0.99 | 0.88 | 0.08 |
| TP10 | 10μM | 0.90 | 1.14 | 1.05 | 0.09 |
|  | 50 μM | 0.83 | 0.95 | 0.90 | 0.05 |
|  | 100 μM | 0.80 | 0.90 | 0.84 | 0.04 |

Table S12. Griess test results in SH-SY5Y cell culture pre-incubated with supernatant from THP-1 cell culture.

| Compounds | Concentration | Min | Max | Mean | SD |
| --- | --- | --- | --- | --- | --- |
| Positive Control | - | 0.93 | 1.03 | 1.00 | 0.04 |
| Negative control | Co-culture | 1.19 | 1.30 | 1.23 | 0.04 |
| TP1 | 10μM | 0.97 | 1.09 | 1.03 | 0.05 |
|  | 50 μM | 0.81 | 0.98 | 0.91 | 0.06 |
|  | 100 μM | 0.74 | 0.88 | 0.80 | 0.06 |
| TP4 | 10μM | 0.98 | 1.12 | 1.04 | 0.06 |
|  | 50 μM | 0.85 | 1.02 | 0.94 | 0.06 |
|  | 100 μM | 0.77 | 0.92 | 0.83 | 0.06 |
| TP5 | 10μM | 0.96 | 1.12 | 1.02 | 0.06 |
|  | 50 μM | 0.87 | 1.03 | 0.96 | 0.06 |
|  | 100 μM | 0.73 | 0.90 | 0.82 | 0.06 |
| TP6 | 10μM | 1.09 | 1.20 | 1.16 | 0.05 |
|  | 50 μM | 0.96 | 1.12 | 1.03 | 0.06 |
|  | 100 μM | 0.92 | 1.06 | 0.98 | 0.06 |
| TP7 | 10μM | 1.09 | 1.28 | 1.18 | 0.07 |
|  | 50 μM | 0.97 | 1.16 | 1.08 | 0.07 |
|  | 100 μM | 0.85 | 1.03 | 0.96 | 0.07 |
| TP8 | 10μM | 0.79 | 0.98 | 0.91 | 0.07 |
|  | 50 μM | 0.78 | 0.89 | 0.84 | 0.04 |
|  | 100 μM | 0.73 | 0.92 | 0.80 | 0.07 |
| TP9 | 10μM | 0.87 | 1.04 | 0.98 | 0.07 |
|  | 50 μM | 0.84 | 1.04 | 0.96 | 0.07 |
|  | 100 μM | 0.80 | 0.99 | 0.89 | 0.07 |
| TP10 | 10μM | 0.87 | 1.05 | 0.96 | 0.07 |
|  | 50 μM | 0.77 | 0.94 | 0.87 | 0.07 |
|  | 100 μM | 0.74 | 0.95 | 0.81 | 0.08 |

# FHA assay results

Table S9. FHA test results in SH-SY5Y cell culture

| Compounds | Concentration | Min | Max | Mean | SD |
| --- | --- | --- | --- | --- | --- |
| Positive Control | - | 0.95 | 1.06 | 1.00 | 0.05 |
| TP1 | 10μM | 1.00 | 1.09 | 1.04 | 0.03 |
|  | 50 μM | 0.99 | 1.08 | 1.05 | 0.03 |
|  | 100 μM | 0.96 | 1.07 | 1.02 | 0.04 |
| TP4 | 10μM | 1.02 | 1.10 | 1.07 | 0.03 |
|  | 50 μM | 0.98 | 1.07 | 1.03 | 0.04 |
|  | 100 μM | 0.99 | 1.02 | 1.01 | 0.02 |
| TP5 | 10μM | 0.98 | 1.02 | 1.01 | 0.02 |
|  | 50 μM | 1.02 | 1.11 | 1.06 | 0.04 |
|  | 100 μM | 1.03 | 1.10 | 1.06 | 0.03 |
| TP6 | 10μM | 0.98 | 1.09 | 1.04 | 0.04 |
|  | 50 μM | 1.01 | 1.08 | 1.04 | 0.03 |
|  | 100 μM | 1.02 | 1.08 | 1.05 | 0.03 |
| TP7 | 10μM | 0.99 | 1.04 | 1.01 | 0.02 |
|  | 50 μM | 1.01 | 1.04 | 1.02 | 0.01 |
|  | 100 μM | 1.03 | 1.05 | 1.04 | 0.01 |
| TP8 | 10μM | 0.96 | 1.05 | 1.01 | 0.03 |
|  | 50 μM | 0.94 | 1.00 | 0.97 | 0.02 |
|  | 100 μM | 0.94 | 1.01 | 0.98 | 0.03 |
| TP9 | 10μM | 0.97 | 1.00 | 0.98 | 0.01 |
|  | 50 μM | 0.95 | 1.00 | 0.97 | 0.02 |
|  | 100 μM | 0.98 | 1.02 | 1.00 | 0.02 |
| TP10 | 10μM | 0.99 | 1.01 | 1.00 | 0.01 |
|  | 50 μM | 0.95 | 1.00 | 0.98 | 0.02 |
|  | 100 μM | 0.95 | 1.01 | 0.98 | 0.02 |

Table S10. FHA test results in SH-SY5Y cell culture pre-incubated with 5 μg/ml LPS.

| Compounds | Concentration | Min | Max | Mean | SD |
| --- | --- | --- | --- | --- | --- |
| Positive Control | - | 0.95 | 1.06 | 1.00 | 0.05 |
| Negative control | 5 μg/ml LPS | 1.91 | 2.07 | 2.00 | 0.07 |
| TP1 | 10μM | 1.16 | 1.29 | 1.21 | 0.05 |
|  | 50 μM | 1.08 | 1.21 | 1.14 | 0.05 |
|  | 100 μM | 0.87 | 0.97 | 0.92 | 0.05 |
| TP4 | 10μM | 1.04 | 1.14 | 1.09 | 0.05 |
|  | 50 μM | 0.97 | 1.08 | 1.01 | 0.05 |
|  | 100 μM | 0.90 | 1.03 | 0.95 | 0.05 |
| TP5 | 10μM | 1.66 | 1.78 | 1.70 | 0.05 |
|  | 50 μM | 1.44 | 1.55 | 1.51 | 0.05 |
|  | 100 μM | 1.45 | 1.58 | 1.52 | 0.05 |
| TP6 | 10μM | 1.62 | 1.76 | 1.69 | 0.05 |
|  | 50 μM | 1.41 | 1.57 | 1.49 | 0.06 |
|  | 100 μM | 1.21 | 1.34 | 1.29 | 0.06 |
| TP7 | 10μM | 1.78 | 1.97 | 1.87 | 0.07 |
|  | 50 μM | 1.39 | 1.56 | 1.49 | 0.07 |
|  | 100 μM | 1.22 | 1.40 | 1.29 | 0.07 |
| TP8 | 10μM | 1.02 | 1.18 | 1.09 | 0.06 |
|  | 50 μM | 1.00 | 1.19 | 1.09 | 0.07 |
|  | 100 μM | 1.01 | 1.15 | 1.08 | 0.07 |
| TP9 | 10μM | 1.46 | 1.63 | 1.54 | 0.06 |
|  | 50 μM | 1.37 | 1.52 | 1.46 | 0.06 |
|  | 100 μM | 1.08 | 1.24 | 1.17 | 0.07 |
| TP10 | 10μM | 1.33 | 1.46 | 1.39 | 0.06 |
|  | 50 μM | 1.12 | 1.26 | 1.19 | 0.06 |
|  | 100 μM | 1.04 | 1.21 | 1.12 | 0.07 |

Table S11. FHA test results in SH-SY5Y cell culture pre-incubated with 50 μg/ml LPS.

| Compounds | Concentration | Min | Max | Mean | SD |
| --- | --- | --- | --- | --- | --- |
| Positive Control | - | 0.95 | 1.06 | 1.00 | 0.05 |
| Negative control | 50 μg/ml LPS | 1.91 | 2.06 | 1.98 | 0.06 |
| TP1 | 10μM | 1.10 | 1.14 | 1.12 | 0.02 |
|  | 50 μM | 1.00 | 1.06 | 1.03 | 0.03 |
|  | 100 μM | 0.95 | 1.07 | 1.01 | 0.05 |
| TP4 | 10μM | 1.08 | 1.13 | 1.10 | 0.02 |
|  | 50 μM | 1.00 | 1.04 | 1.02 | 0.02 |
|  | 100 μM | 0.97 | 1.04 | 1.01 | 0.03 |
| TP5 | 10μM | 1.34 | 1.42 | 1.37 | 0.03 |
|  | 50 μM | 1.28 | 1.32 | 1.30 | 0.02 |
|  | 100 μM | 1.14 | 1.25 | 1.21 | 0.04 |
| TP6 | 10μM | 1.51 | 1.60 | 1.56 | 0.03 |
|  | 50 μM | 1.33 | 1.39 | 1.37 | 0.03 |
|  | 100 μM | 1.26 | 1.32 | 1.29 | 0.02 |
| TP7 | 10μM | 1.93 | 2.06 | 2.00 | 0.06 |
|  | 50 μM | 1.35 | 1.41 | 1.39 | 0.02 |
|  | 100 μM | 1.26 | 1.30 | 1.28 | 0.02 |
| TP8 | 10μM | 0.97 | 1.07 | 1.03 | 0.04 |
|  | 50 μM | 0.96 | 1.05 | 1.00 | 0.03 |
|  | 100 μM | 0.90 | 1.03 | 0.97 | 0.06 |
| TP9 | 10μM | 1.40 | 1.50 | 1.46 | 0.04 |
|  | 50 μM | 1.25 | 1.48 | 1.40 | 0.09 |
|  | 100 μM | 1.20 | 1.37 | 1.30 | 0.07 |
| TP10 | 10μM | 1.43 | 1.47 | 1.45 | 0.02 |
|  | 50 μM | 1.37 | 1.44 | 1.39 | 0.03 |
|  | 100 μM | 1.24 | 1.37 | 1.28 | 0.06 |

Table S12. FHA test results in SH-SY5Y cell culture pre-incubated with supernatant from THP-1 cell culture.

| Compounds | Concentration | Min | Max | Mean | SD |
| --- | --- | --- | --- | --- | --- |
| Positive Control | - | 0.95 | 1.06 | 1.00 | 0.05 |
| Negative control | Co-culture | 1.72 | 1.86 | 1.78 | 0.05 |
| TP1 | 10μM | 1.08 | 1.29 | 1.17 | 0.08 |
|  | 50 μM | 1.06 | 1.15 | 1.09 | 0.04 |
|  | 100 μM | 0.95 | 1.08 | 1.03 | 0.05 |
| TP4 | 10μM | 1.01 | 1.11 | 1.06 | 0.04 |
|  | 50 μM | 0.97 | 1.11 | 1.04 | 0.07 |
|  | 100 μM | 0.99 | 1.04 | 1.02 | 0.02 |
| TP5 | 10μM | 1.26 | 1.29 | 1.27 | 0.01 |
|  | 50 μM | 1.23 | 1.36 | 1.30 | 0.05 |
|  | 100 μM | 1.11 | 1.23 | 1.18 | 0.05 |
| TP6 | 10μM | 1.73 | 1.82 | 1.76 | 0.04 |
|  | 50 μM | 1.64 | 1.73 | 1.68 | 0.05 |
|  | 100 μM | 1.54 | 1.62 | 1.59 | 0.03 |
| TP7 | 10μM | 1.83 | 1.91 | 1.88 | 0.04 |
|  | 50 μM | 1.76 | 1.83 | 1.79 | 0.03 |
|  | 100 μM | 1.61 | 1.75 | 1.68 | 0.06 |
| TP8 | 10μM | 1.02 | 1.19 | 1.09 | 0.07 |
|  | 50 μM | 0.94 | 0.98 | 0.97 | 0.02 |
|  | 100 μM | 0.90 | 0.93 | 0.92 | 0.02 |
| TP9 | 10μM | 1.66 | 1.70 | 1.68 | 0.02 |
|  | 50 μM | 1.55 | 1.64 | 1.60 | 0.03 |
|  | 100 μM | 1.28 | 1.40 | 1.34 | 0.05 |
| TP10 | 10μM | 1.69 | 1.75 | 1.71 | 0.03 |
|  | 50 μM | 1.58 | 1.67 | 1.62 | 0.04 |
|  | 100 μM | 1.34 | 1.44 | 1.41 | 0.04 |

# COX activity

Table S13. COX activity test results in SH-SY5Y cells pre-incubated with 50 μg/ml

| Compounds |  | Min | Max | Mean | SD |
| --- | --- | --- | --- | --- | --- |
| Control |  | 167.30 | 217.59 | 197.45 | 26.60 |
| TP1 | Total COX | 113.95 | 170.95 | 137.71 | 29.66 |
|  | COX-1 | 89.93 | 104.81 | 99.03 | 7.98 |
|  | COX-2 | 94.04 | 147.03 | 117.04 | 27.18 |
| TP4 | Total COX | 51.24 | 86.63 | 71.67 | 18.32 |
|  | COX-1 | 40.17 | 90.57 | 61.39 | 26.13 |
|  | COX-2 | 68.42 | 109.71 | 86.98 | 20.96 |
| TP5 | Total COX | 54.62 | 97.89 | 78.74 | 22.06 |
|  | COX-1 | 32.62 | 66.57 | 53.86 | 18.51 |
|  | COX-2 | 107.46 | 123.30 | 115.12 | 7.93 |
| TP6 | Total COX | 50.69 | 92.52 | 77.21 | 23.05 |
|  | COX-1 | 46.57 | 65.12 | 53.96 | 9.83 |
|  | COX-2 | 107.28 | 122.89 | 115.37 | 7.82 |
| TP7 | Total COX | 77.39 | 101.64 | 87.33 | 12.70 |
|  | COX-1 | 53.95 | 79.98 | 67.03 | 13.01 |
|  | COX-2 | 124.52 | 129.88 | 127.55 | 2.75 |
| TP8 | Total COX | 83.46 | 128.65 | 99.57 | 25.23 |
|  | COX-1 | 74.73 | 106.59 | 89.55 | 16.05 |
|  | COX-2 | 135.11 | 160.69 | 146.79 | 12.93 |
| TP9 | Total COX | 85.45 | 111.83 | 98.22 | 13.21 |
|  | COX-1 | 14.09 | 44.43 | 30.39 | 15.30 |
|  | COX-2 | 118.63 | 147.09 | 133.49 | 14.27 |
| TP10 | Total COX | 56.02 | 90.27 | 74.24 | 17.23 |
|  | COX-1 | 7.74 | 38.28 | 21.93 | 15.39 |
|  | COX-2 | 92.21 | 126.11 | 112.28 | 17.79 |
