# Supplementary material for: Effect of tricyclic 1,2-thiazine derivatives in neuroinflammation induced by preincubation with lipopolysaccharide or coculturing with microglia-like cells
Source: Pharmacol Rep. 2022 Sep 21;74(5):890–908. doi: 10.1007/s43440-022-00414-8 (PMC9584986; doi:10.1007/s43440-022-00414-8)
Supplement: Supplementary file 2 — Supplementary file2 (PDF 376 kb) [file 43440_2022_414_MOESM2_ESM.pdf]

Control without LPS or THP-1 supernatant	Control with 5 μg/ml LPS	Control with 50 μg/ml LPS	Control with supernatant from THP-1 cells
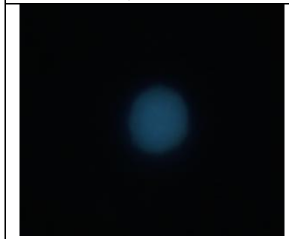	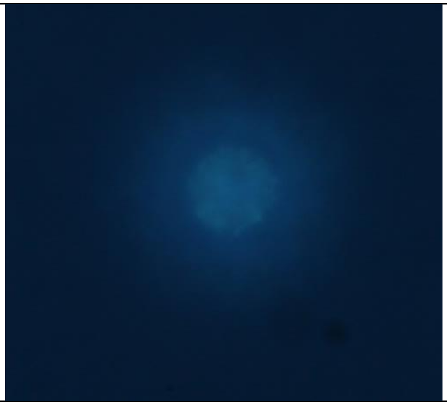	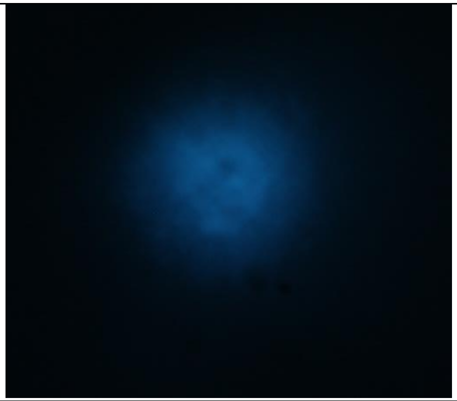	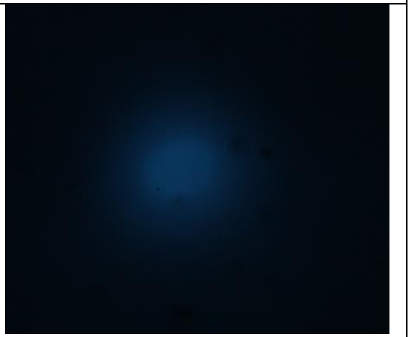

Cells pre-incubated with 5 µg/ml LPS			
Compounds	10 µM	50 µM	100 µM
TP1	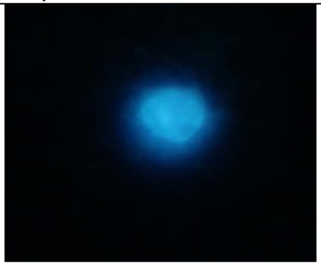	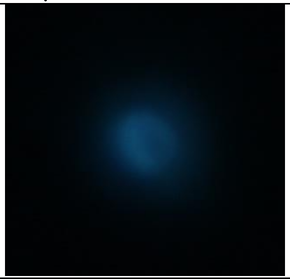	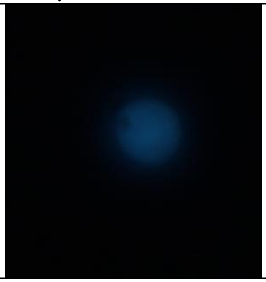
TP4	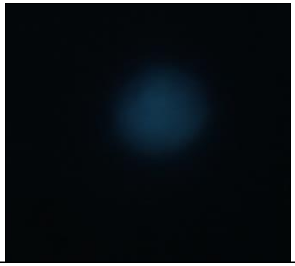	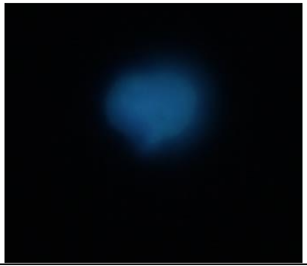	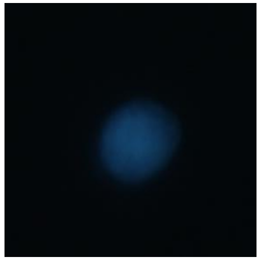
TP5	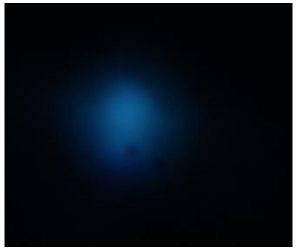	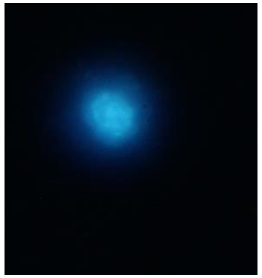	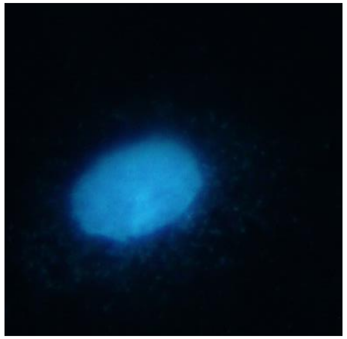

Cells pre-incubated with 5 µg/ml LPS			
Compounds	10 µM	50 µM	100 µM
TP6	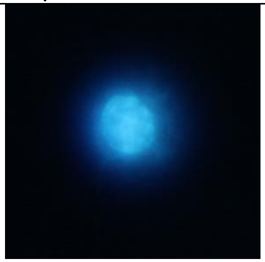	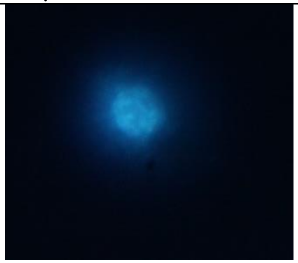	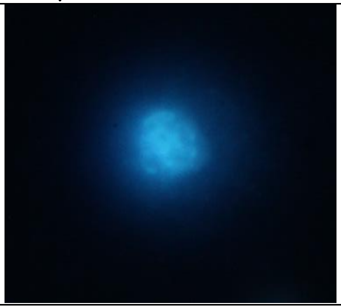
TP7	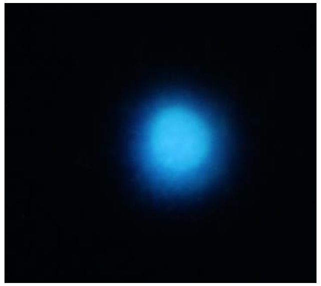	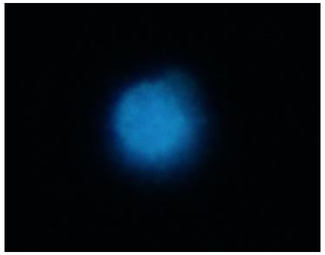	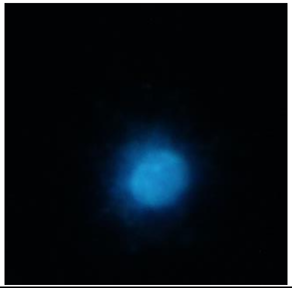
TP8	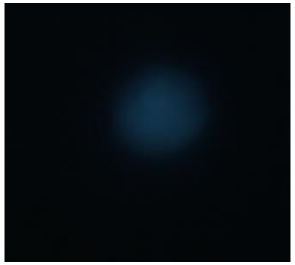	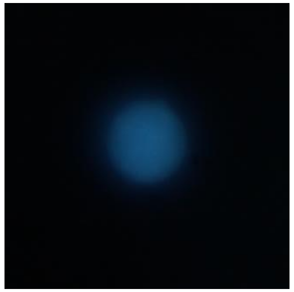	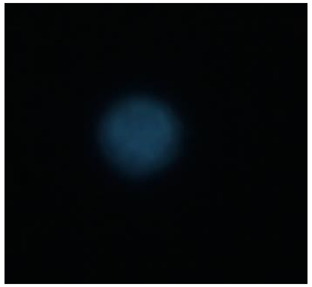

Cells pre-incubated with 5 µg/ml LPS			
Compounds	10 µM	50 µM	100 µM
TP9	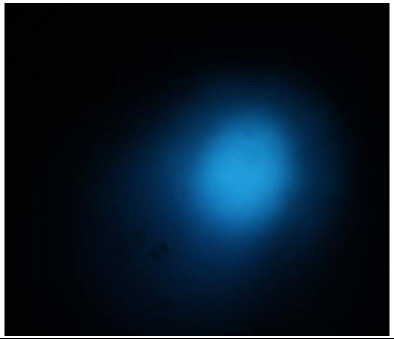	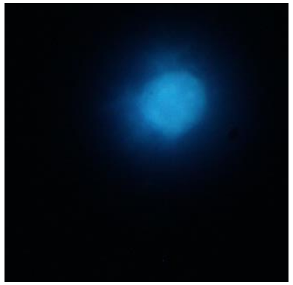	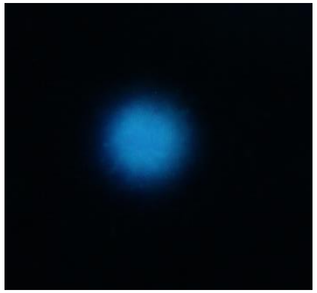
TP10	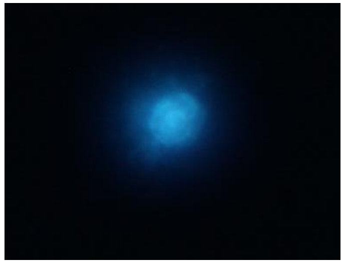	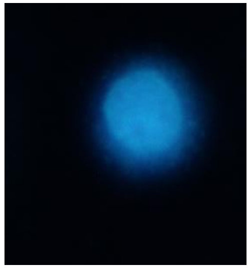	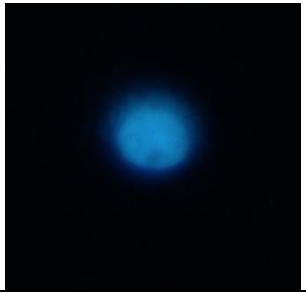

Cells pre-incubated with 50 µg/ml LPS			
Compounds	10 µM	50 µM	100 µM
TP1	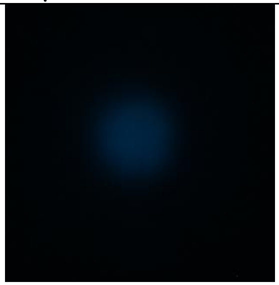	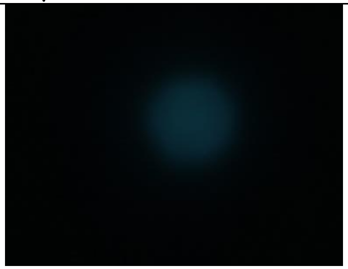	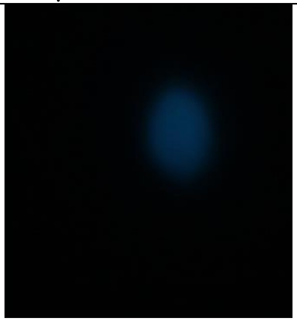
TP4	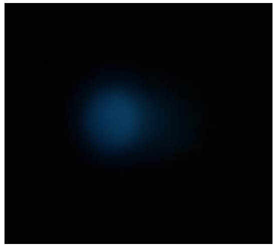	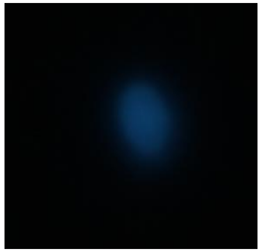	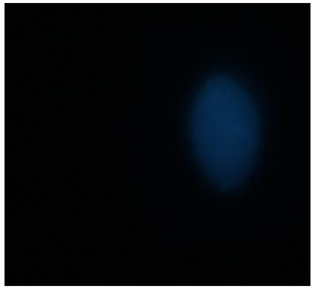
TP5	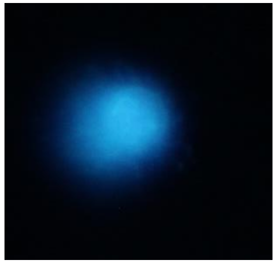	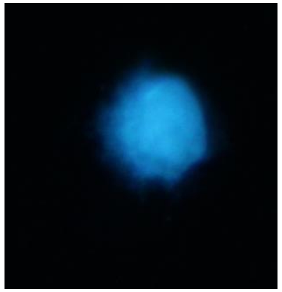	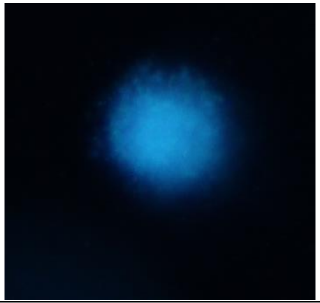

Cells pre-incubated with 50 µg/ml LPS			
Compounds	10 µM	50 µM	100 µM
TP6	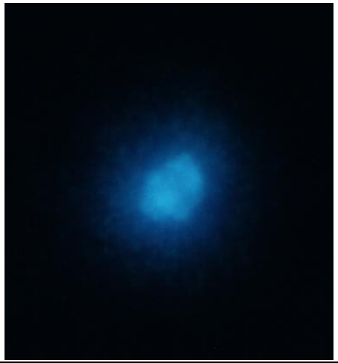	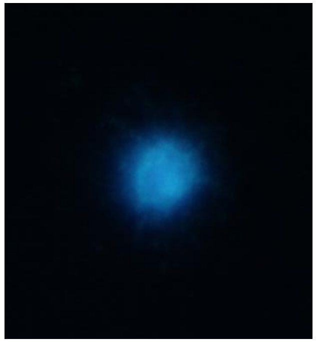	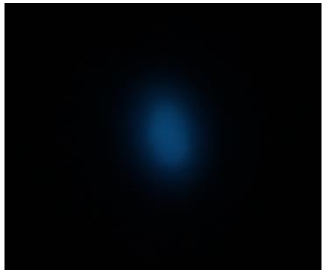
TP7	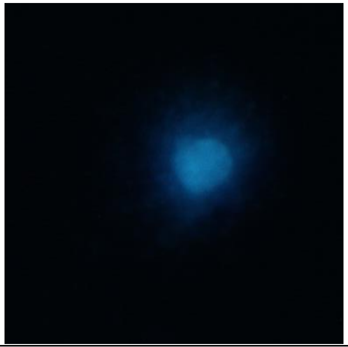	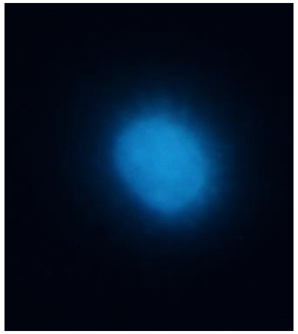	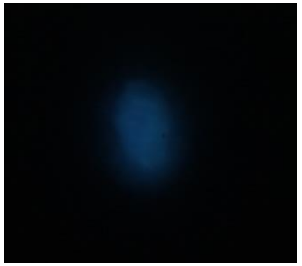
TP8	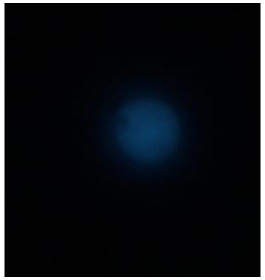	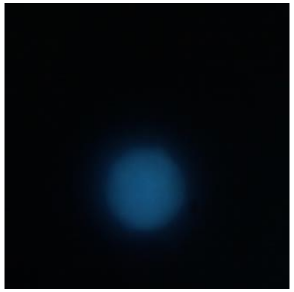	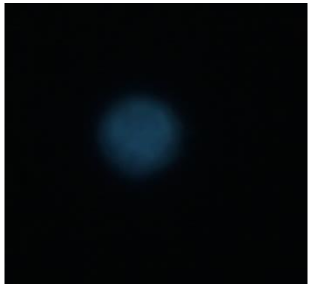

Cells pre-incubated with 50 µg/ml LPS			
Compounds	10 µM	50 µM	100 µM
TP9	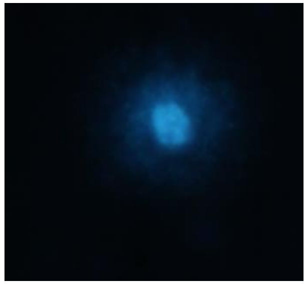	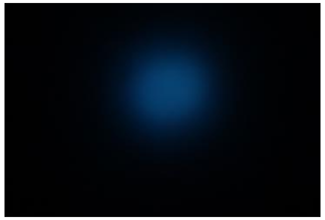	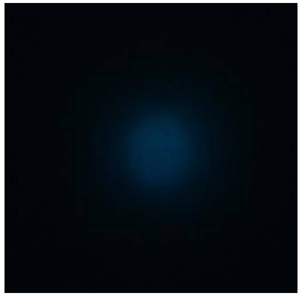
TP10	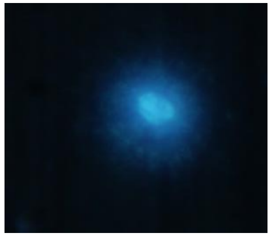	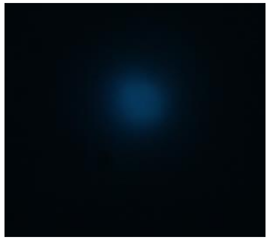	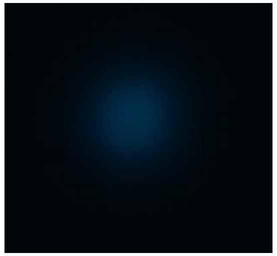

Cells pre-incubated with THP-1 supernatant			
Compounds	10 μM	50 μM	100 μM
TP1	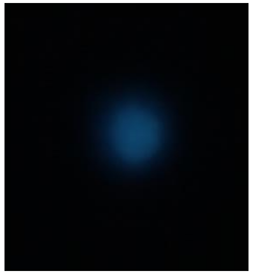	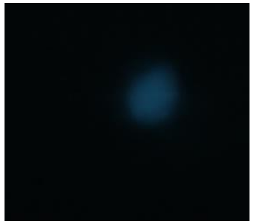	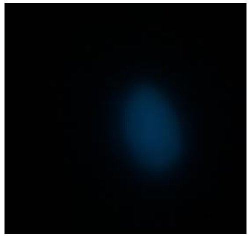
TP4	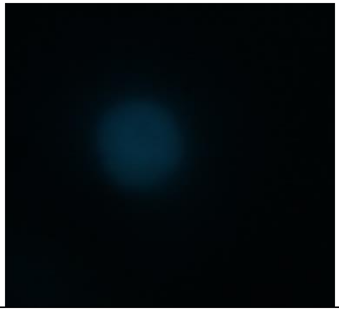	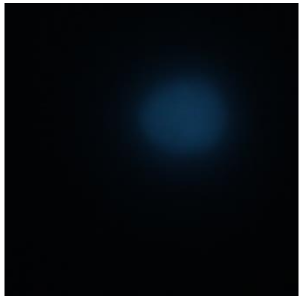	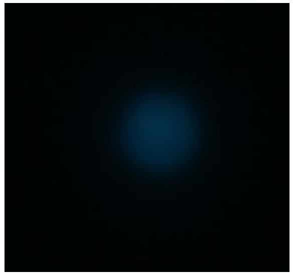
TP5	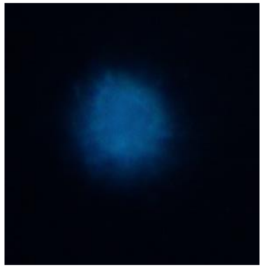	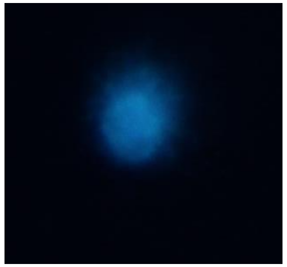	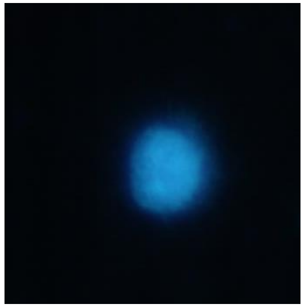

Cells pre-incubated with THP-1 supernatant			
Compounds	10 μM	50 μM	100 μM
TP6	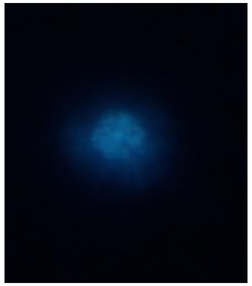	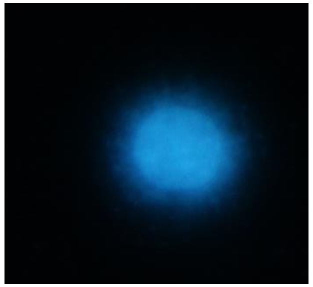	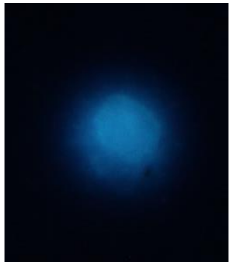
TP7	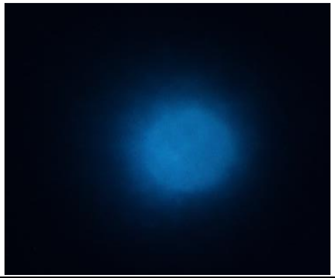	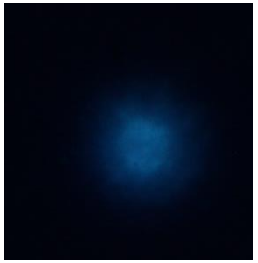	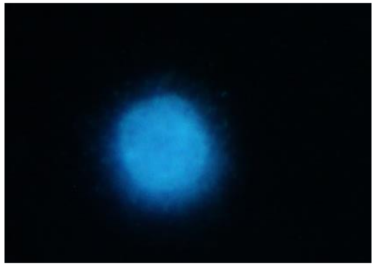
TP8	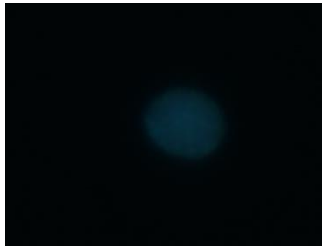	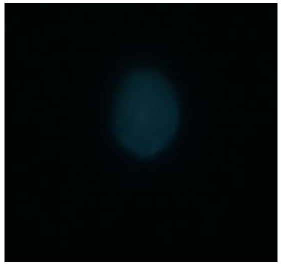	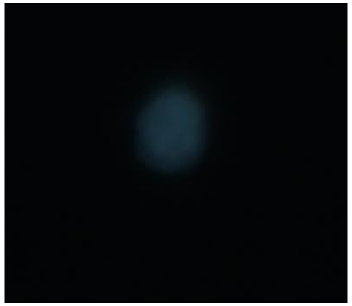

Cells pre-incubated with THP-1 supernatant			
Compounds	10 μM	50 μM	100 μM
TP9	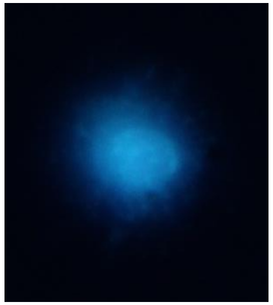	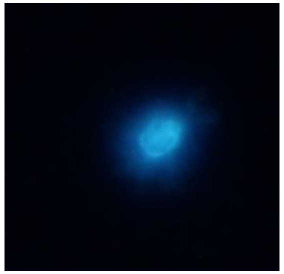	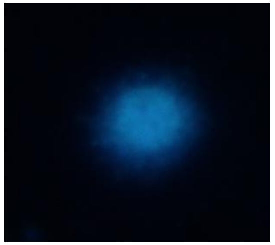
TP10	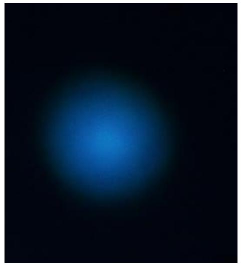	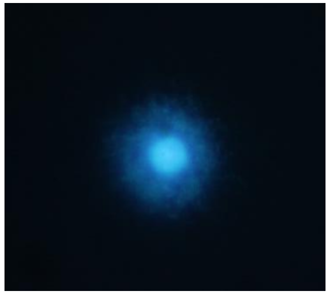	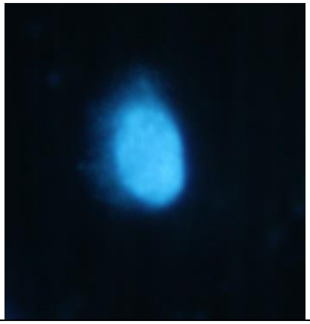
