# Supplementary material for: Effect of tricyclic 1,2-thiazine derivatives in neuroinflammation induced by preincubation with lipopolysaccharide or coculturing with microglia-like cells
Source: Pharmacol Rep. 2022 Sep 21;74(5):890–908. doi: 10.1007/s43440-022-00414-8 (PMC9584986; doi:10.1007/s43440-022-00414-8)
Supplement: Supplementary file 4 — Supplementary file4 (DOCX 718 kb) [file 43440_2022_414_MOESM4_ESM.docx]

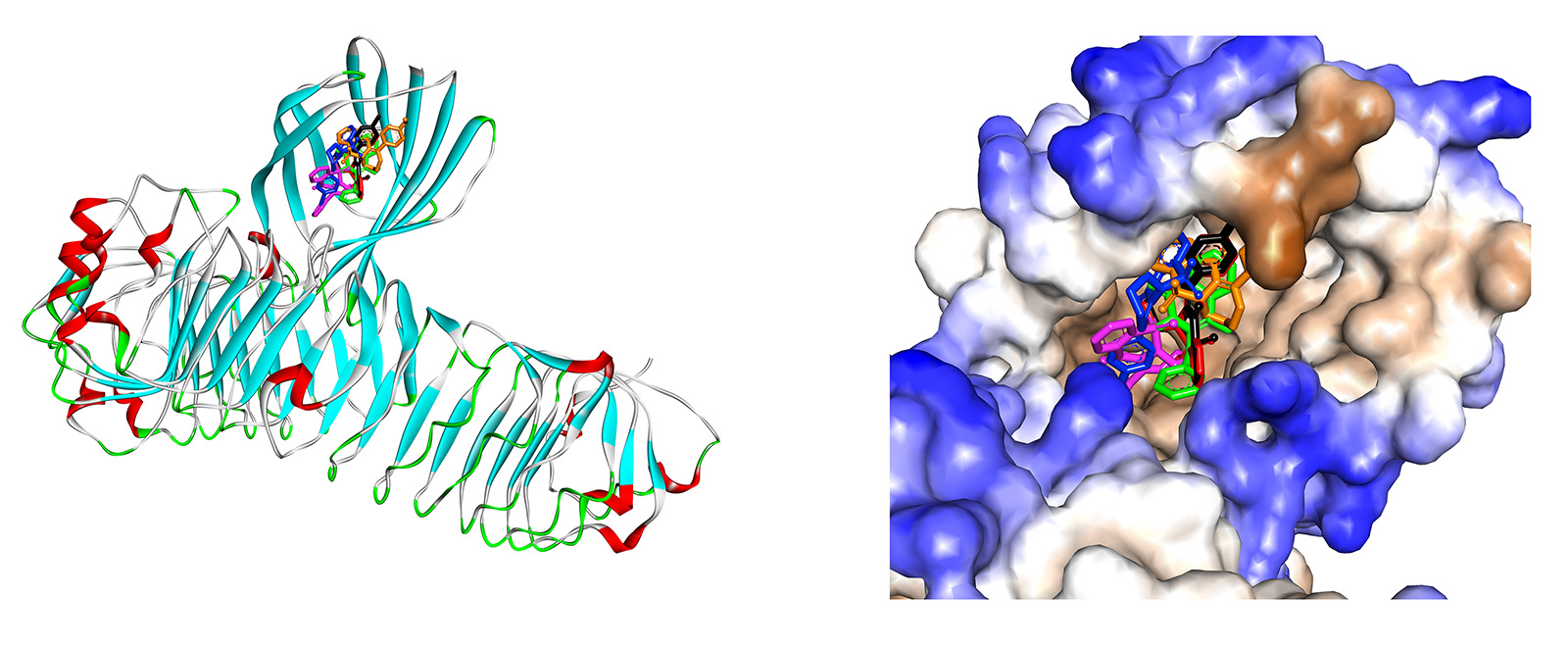


Figure 1S. The docked pose of TP1 (green), TP5 (red), TP6 (blue), TP7 (black), TP9 (orange), TP10 (pink)into pocket site of TLR4/MD-2 complex.


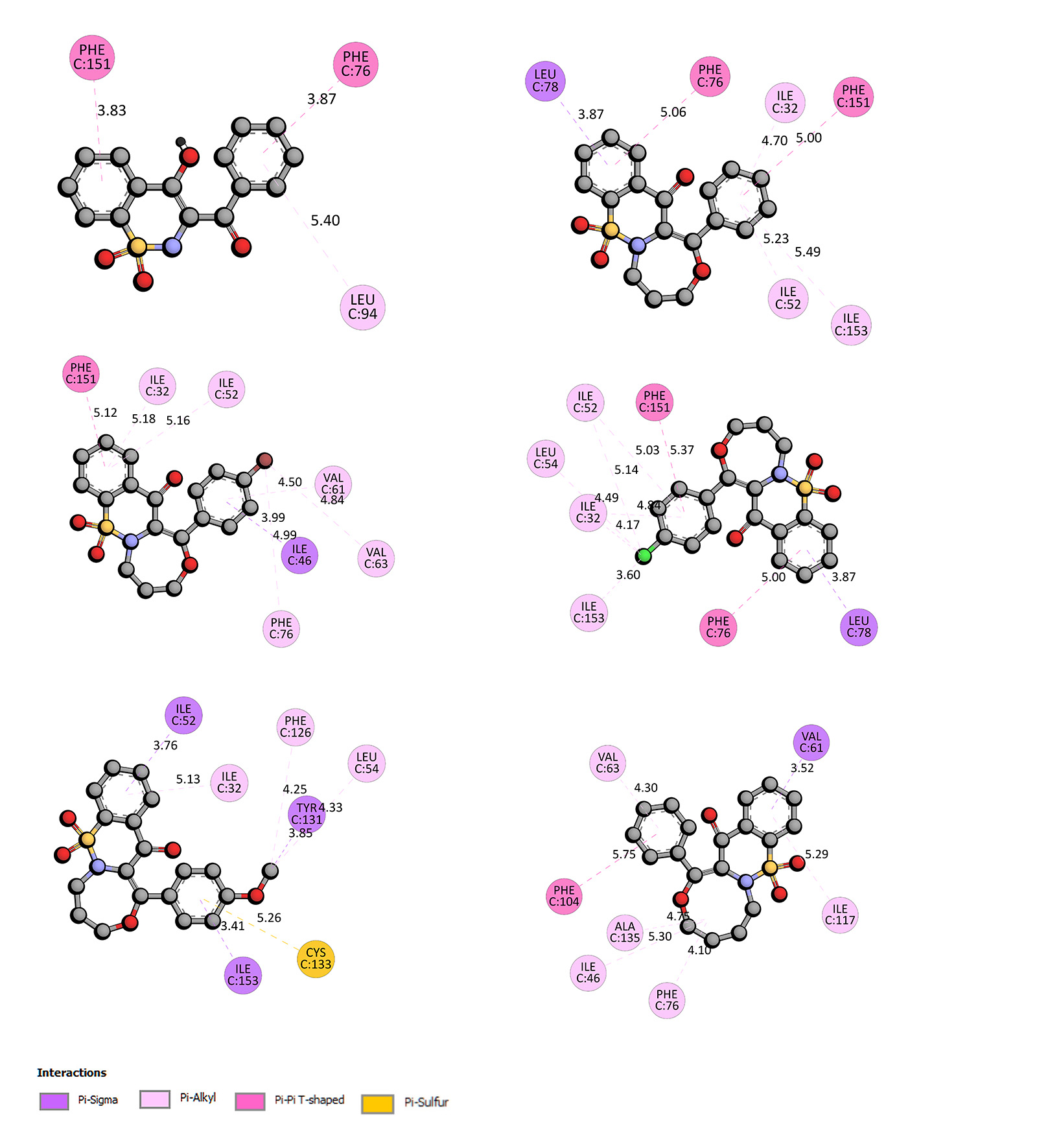


Figure 2S. The 2D plot of interaction TP1, TP5, TP6, TP7, TP9, and TP10 with TLR4/MD-2 complex.
